# The Impact of a Limited Field-of-View on Computed Hemodynamics in Abdominal Aortic Aneurysms: Evaluating the Feasibility of Completing Ultrasound Segmentations with Parametric Geometries

**DOI:** 10.1007/s10439-022-03133-6

**Published:** 2023-01-28

**Authors:** Judith Fonken, Esther Maas, Arjet Nievergeld, Marc van Sambeek, Frans van de Vosse, Richard Lopata

**Affiliations:** 1 Photoacoustics & Ultrasound Laboratory Eindhoven (PULS/e), Department of Biomedical Engineering, Eindhoven University of Technology, Eindhoven, The Netherlands; 2 Department of Surgery, Catharina Hospital Eindhoven, Eindhoven, The Netherlands; 3 Cardiovascular Biomechanics, Department of Biomechanical Engineering, Eindhoven University of Technology, Eindhoven, The Netherlands

**Keywords:** Abdominal aortic aneurysm, Hemodynamics, Ultrasound, Computational fluid dynamics, Fluid–structure interaction, Personalized modeling, Field-of-view, Rupture risk assessment

## Abstract

To improve abdominal aortic aneurysm (AAA) rupture risk assessment, a large, longitudinal study on AAA hemodynamics and biomechanics is necessary, using personalized fluid-structure interaction (FSI) modeling. 3-dimensional, time-resolved ultrasound (3D+t US) is the preferred image modality to obtain the patient-specific AAA geometry for such a study, since it is safe, affordable and provides temporal information. However, the 3D+t US field-of-view (FOV) is limited and therefore often fails to capture the inlet and aorto-iliac bifurcation geometry. In this study, a framework was developed to add parametric inlet and bifurcation geometries to the abdominal aortic aneurysm geometry by employing dataset statistics and parameters of the AAA geometry. The impact of replacing the patient-specific inlet and bifurcation geometries, acquired using computed tomography (CT) scans, by parametric geometries was evaluated by examining the differences in hemodynamics (systolic and time-averaged wall shear stress and oscillatory shear index) in the aneurysm region. The results show that the inlet geometry has a larger effect on the AAA hemodynamics (median differences of 7.5 to 18.8%) than the bifurcation geometry (median differences all below 1%). Therefore, it is not feasible to replace the patient-specific inlet geometry by a generic one. Future studies should investigate the possibilities of extending the proximal FOV of 3D+t US. However, this study did show the feasibility of adding a parametric bifurcation geometry to the aneurysm geometry. After extending the proximal FOV, the obtained framework can be used to extract AAA geometries from 3D+t US for FSI simulations, despite the absence of the bifurcation geometry.

**Supplementary Information:**

The online version of this article (10.1007/s10439-022-03133-6) contains supplementary material, which is available to authorized users.

## Introduction

An abdominal aortic aneurysm (AAA), a local dilation of the infrarenal aorta, is often asymptomatic, but can expand until rupture occurs, which is accompanied by an overall mortality of 80%.^14,21^ Surgical repair of AAAs can be performed to prevent rupture, but is not without risks either.^14^ Therefore, the patient’s risk of rupture is assessed and monitored over time. In current clinical guidelines, the rupture risk is estimated using the maximum diameter and growth rate of the aneurysm, which is based on randomized clinical trials.^6,15^ However, it is suggested that wall mechanics and hemodynamics will provide better risk indicators.^14,21^

In numerous previous studies, computational solid stress (CSS) and computational fluid dynamic (CFD) models have been employed to examine, among others, (peak) wall stress and wall shear stress (WSS), respectively. Peak wall stress is believed to be an important parameter in predicting aneurysm rupture, whereas low, disturbed WSS is believed to lead to formation of intraluminal thrombus (ILT).^2,3,5,12,16,21,29,32,36,37^ The effect of ILT on AAA development is still under debate. Some studies suggest that the presence of ILT lowers the AAA wall stress, possibly preventing AAA rupture.^27,32,36,37^ However, other studies argue that ILT leads to weaking of the wall, since the transport of solutes and the interaction between the vessel wall and hemodynamic forces is disrupted.^33,37^

*In vivo*, the deformation of the AAA wall is influenced by the hemodynamics in the AAA and vice versa. To incorporate this interaction in AAA simulations, fluid-structure interaction (FSI) models need to be employed.^18,21^ In CSS simulations, an uniform pressure is applied to the AAA wall, whereas a pressure gradient in proximal-distal direction is observed in FSI simulations. As long as the pressure gradient is small, insignificant differences were observed between the CSS and FSI simulations.^18^ In CFD simulations, the wall is assumed to be rigid, which results in an underestimation of vortex formation and overestimation of WSS compared to FSI simulations.^18^

A large, longitudinal study is required to develop a better understanding and prediction of AAA development, growth and rupture risk. However, since both wall mechanics and hemodynamics are highly dependent on AAA geometry, a patient-specific assessment is necessary.^5,21^ Computed tomography (CT) is the golden standard to obtain patient-specific AAA geometries. This poses a problem, since the use of contrast agents and radiation hampers frequent use.^21^ Since previous FSI studies used either idealized AAA geometries^18,22,23^ or a small set of CT-derived geometries,^10,24,34,36^ no longitudinal studies employing FSI simulations are executed yet. For such a study, time-resolved 3-dimensional ultrasound (3D+t US) is the preferred image modality to extract the patient-specific geometry, since it is safe, fast and affordable. Additionally, 3D+t US contains temporal geometric and functional information, and ultrasound is already used in the current clinical workflow.^30^

However, the use of 3D+t US data in acquiring patient-specific geometries for (FSI) simulations is hindered by its limitations with respect to CT. Firstly, 3D+t US is limited in terms of contrast. Nevertheless, recent improvements in segmentation methods allow for the use of 3D+t US data in CSS and FSI models,^13,29,30^ showcasing good correspondence between 3D+t US-based and CT-based segmentations and resulting wall stresses.^29^ In these models, the temporal geometric information obtained from 3D+t US can even be used to estimate the patient-specific wall stiffness, as demonstrated by van Disseldorp *et al*.^30^

**FIGURE 1:**
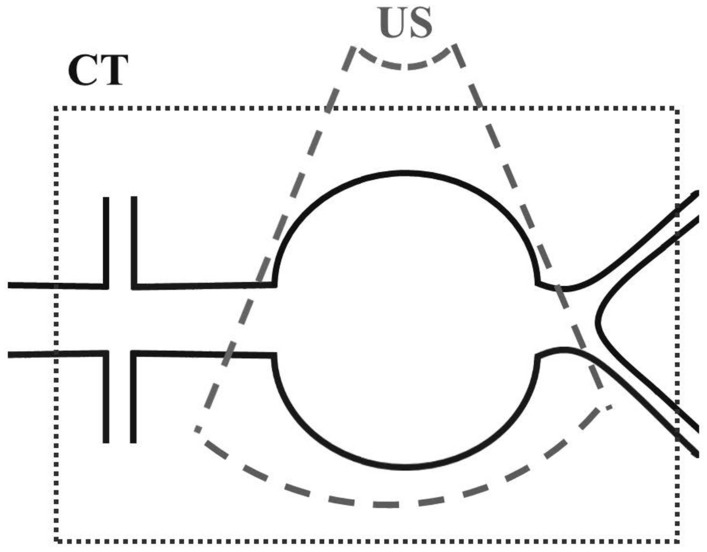
Graphical illustration of an abdominal aortic aneurysm (AAA) with the field-of-view of computed tomography (CT) (black dotted line) and that corresponding to ultrasound (US) imaging (gray striped line). Figure adopted from Disseldorp *et al*.^28^

Secondly, due to the limited field-of-view of US compared to CT (Fig. 1), the inlet (defined as the region between renal arteries and aneurysm) and aorto-iliac bifurcation geometry often cannot be obtained with a single 3D+t US acquisition. Additionally, the bifurcation is hard to detect with ultrasound, due to the depth and tortuosity of the iliac arteries.^28^ It has been shown that the limited FOV and the absence of the bifurcation does not significantly influence the numerical assessment of wall mechanics in the AAA region, as long as the shoulders of the aneurysm are captured in the FOV.^28^ In the fluid domain however, previous studies have shown that both the inlet and bifurcation angle affect the peak WSS, whereas only the inlet angle has a significant effect on the blood flow patterns.^11,17,35^ However, idealized AAA geometries were employed and no other parameters, such as the length of the inlet, the iliac radii, and the distance between aneurysm region and bifurcation, were taken into account in these studies. Furthermore, for the increase in bifurcation angle, the major increase in WSS occurred in the iliac arteries. In the AAA region, only minor differences in WSS were observed for different iliac angulations.^35^

The aim of this study is to evaluate the feasibility of replacing the patient-specific inlet and bifurcation by parametric geometries. To this end, patient-specific geometries were obtained from CT scans of AAA patients. These CT-based geometries were limited in FOV to resemble the 3D+t US FOV and employed to obtain geometries with parametric inlet or bifurcation regions. For each patient, simulations for the patient-specific, parametric inlet and parametric bifurcation geometries were executed and the hemodynamics in the aneurysm region were compared to quantify the influence of replacing the patient-specific inlet or bifurcation geometry by a parametric one.

## Materials and Methods

**FIGURE 2:**
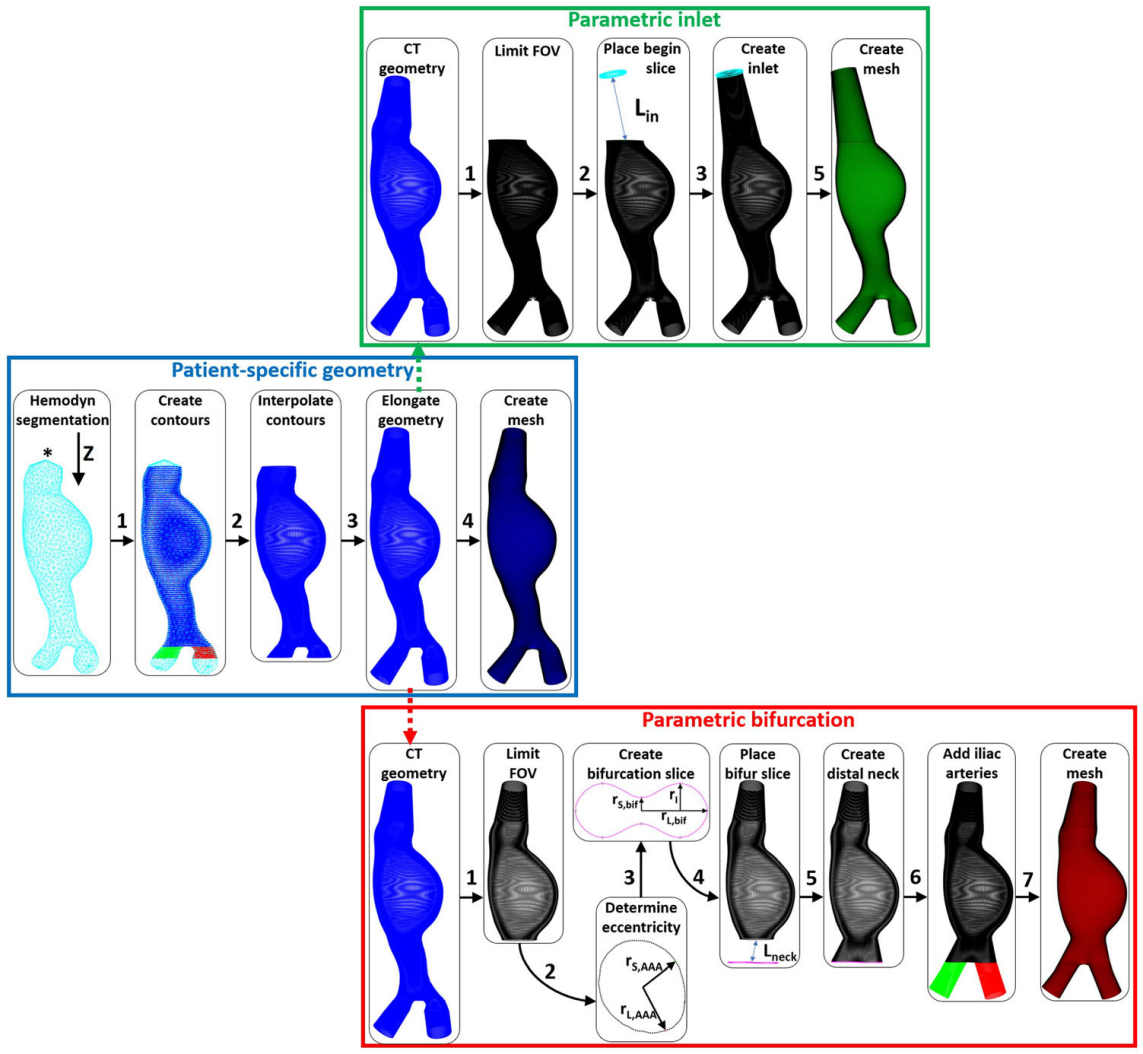
Framework to create patient-specific (middle, blue), parametric inlet (top, green) and parametric bifurcation (bottom, red) geometries for a single patient. The asterisk in the Hemodyn segmentation indicates the termination node.

In a collaborative study with the Catharina Hospital in Eindhoven, pre-operative CT scans of 16 AAA patients were acquired using a Philips iCT 256-slice (*n* = 11) or a Philips Brilliance 64-channel (*n* = 5) scanner as part of the normal clinical workflow. The CT scans had an in-plane resolution of 0.6–0.8 by 0.6–0.8 mm and a slice thickness of 3 mm. Furthermore, blood pressure measurements were obtained for all patients during each clinical visit. The study was approved by the local ethics committee, and all patients gave their written informed consent. A workflow was developed to generate both CT-derived, patient-specific geometries (“Patient-Specific Geometry” section) as well as geometries with a parametric inlet (“Parametric Inlet Geometry” section) and a parametric bifurcation (“ Parametric bifurcation geometry” section), as shown in Fig. 2.

### Patient-Specific Geometry

Each patient-specific AAA geometry was segmented semi-automatically from the CT data using the Hemodyn software package developed by the Eindhoven University of Technology (Eindhoven, the Netherlands) and Philips Medical Systems (Best, the Netherlands).^4,20^ In summary, a starting point proximal to the AAA and end points in both iliac arteries were selected manually, after which the aortic centerline was tracked automatically. Subsequently, a 3-dimensional active object was inflated until the AAA inner wall was reached. The automatic segmentation was inspected visually and adjusted if deemed necessary. The faces and vertices describing the Hemodyn inner wall segmentation were exported to MATLAB (R2021b, Matworks Inc., Natick, MA, USA).

The patient-specific workflow (middle in Fig. 2) starts with converting the Hemodyn segmentations into contours by extracting the vertices at specified *Z*-coordinates. The contours were divided in abdominal aorta (AA, blue), left common iliac (L, red), and right common iliac (R, green) contours. In the Hemodyn segmentation, the proximal end of the segmentation was terminated with the initial starting point, leading to contours with erroneous small diameters. Therefore, the end contour was defined as the contour including the node with the smallest *Z*-coordinate connected to the termination node (indicated with an asterisk in Fig. 2). Furthermore, the iliac arteries often move out of the longitudinal (*Z*) plane, due to their high curvature. Hence, the iliac contours were inspected visually and parts were removed when the Hemodyn segmentation moved out of plane.

Subsequently, the contours were interpolated to obtain the desired mesh size of 0.8 mm.^13^ To reduce the effects of the boundary conditions on the results in the AAA region, the segmentation was elongated in the direction of the AAA centerline by 3 and 2 cm (Fig. S1) in proximal and distal direction, respectively, resulting in a circular inlet and outlets perpendicular to the centerline, which are required to prescribe the boundary conditions in the fluid domain. The radius of the inlet equalled 1 cm, whereas the radius of the outlet equalled the radius of the last patient-specific iliac contour. Lastly, the patient-specific surface mesh was obtained by connecting the contours in quadrangular faces. In ten iterations, the mesh was smoothed using curvature flow smoothing.^8^

### Parametric Inlet Geometry

The patient-specific, CT-derived geometry was cropped proximal to the aneurysm region to obtain a FOV that resembles the 3D+t US FOV, by manually selecting the first aneurysm contour. For each patient, the length of the inlet ($L_{{\text{in}}}$) was determined.

Using the median inlet length of the dataset and the limited FOV geometry, a parametric inlet geometry was added, as shown in Fig. 2 (top). A generic inlet radius of 1 cm was used to create a circular inlet contour, which was placed at the desired distance ($L_{{\text{in}}}$) from the last AAA curve, in the direction of the AAA centerline. The inlet geometry was obtained by linear interpolation between the last AAA slice and the inlet slice. Similar to the patient-specific geometry, the surface mesh was obtained by connecting the contours and smoothed using curvature flow smoothing.

### Parametric Bifurcation Geometry

For each patient, the CT-based geometry was used to determine the parameters of the patient-specific bifurcation geometry. The last aortic contour proximal to the iliac arteries is referred to as the bifurcation (bif) contour and its shape approaches a lemniscate curve. The last AAA contour, defined as the last curve belonging to the aneurysm region in distal direction, was selected manually and will later also be used to define the limited FOV. For both the bifurcation contour and the last AAA contour, the short radius ($r_{\text{S}}$) and long radius ($r_{\text{L}}$) were determined by calculating the distance from each point on the contour to the center of the contour, and extracting the $10{\text{th}}$ and $90{\text{th}}$ percentile of all distances, respectively. Subsequently, the eccentricity of the bifurcation and last AAA contour ($e_{\text{bif}}$ and $e_{\text{AAA}}$, respectively) were calculated using:

1 $$e = \sqrt {1 - \frac{{r_{{\text{S}}}^{2} }}{{r_{{\text{L}}}^{2} }}}$$

The region between the last AAA and first bifurcation curve was referred to as the distal neck. Towards the bifurcation curve, the eccentricity and long radius increase and the short radius decreases. The eccentricity and long radius increase rate ($\Delta e$ and $\Delta r_{\text{L}}$, respectively) and the short radius decrease rate ($\Delta r_{\text{S}}$) were calculated using:

2 $$\Delta X = \sqrt {\frac{{X_{{{\text{bif}}}} - X_{{{\text{AAA}}}} }}{{L_{{{\text{neck}}}} }}}$$

With *X* the parameter of interest (*e*, $r_{\text{L}}$ or $r_{\text{S}}$) and $L_{\text{neck}}$ the distal neck length.

For all iliac contours, the radius was determined by calculating the distance from each point on the contour to the center and taking the mean value. Subsequently, the ratio between the left and right iliac radii $(\hbox {R}_{\text{R},\text{L}})$, the ratio between the first and last iliac radii $(\hbox {R}_{1,\text{end}})$, and the ratio between the long radius of the bifurcation curve and the iliac radius $(\hbox {R}_{\text{r}_{\text{L}_\text{bif}}}$,$\hbox {r}_{\text{I}}$) were calculated. Lastly, the length of the iliac arteries $(\hbox {L}_{\text{I}}$) and the bifurcation angle ($\alpha _{\text{R},\text{L}})$, the angle between the left and right iliac centerlines, were determined.

The elongated CT geometry was used as starting point of the workflow to complete the limited FOV geometry with a parametric bifurcation (bottom in Fig. 2). The CT-based geometry was cropped directly distal to the AAA region to resemble the FOV that can be obtained with a 3D US acquisition. The short and long radii and eccentricity of the last AAA contour were determined and subsequently used to calculate the length of the parametric distal neck ($L_{\text{neck}}$), the short and long radii of the bifurcation curve ($r_{\text{S,bif}}$ and $r_{\text{L,bif}}$, respectively) and the iliac radius ($r_{\text{I}}$):

3 $$\begin{aligned} \begin{aligned}&L_{\text{neck}} = \frac{e_{\text{bif}}-e_{\text{AAA}}}{\Delta e}\\&r_{\text{L,bif}} = r_{\text{L,AAA}} + \Delta r_{\text{L}} \cdot L_{\text{neck}}\\&r_{\text{S,bif}} = r_{\text{S,AAA}} - \Delta r_{\text{S}} \cdot L_{\text{neck}}\\&r_{\text{I}} = R_{r_{\text{L,bif}},r_{\text{I}}} \cdot r_{\text{L,bif}} \end{aligned} \end{aligned}$$

These parameters were used to generate an idealized bifurcation slice and to place it at the desired distance from the last AAA curve, in the direction of the AAA centerline. The distal neck was created by linear interpolation between the last AAA slice and bifurcation slice. Subsequently, the iliac arteries were added by assuming an equal angle between iliac and AAA centerline for both left and right common iliac ($\frac{\alpha _{\text{R,L}}}{2}$). Finally, the surface mesh was obtained in a similar fashion as for the patient-specific mesh.

For some geometries, the last AAA contour was located directly proximal to the iliac arteries. In these cases, the length of the distal neck was smaller than the desired mesh size and no distal neck and idealized bifurcation slice were added. The parametric iliac arteries were created directly distal to the last AAA curve, using the long radius of the last AAA curve to obtain the iliac radius.

### Computational Fluid Dynamics Simulations

Since only the differences in hemodynamic properties, not the exact values, were examined in this study, CFD simulations were employed instead of FSI simulations. To verify this approach, for a single patient, CFD and FSI simulations were performed for the patient-specific and parametric bifurcation geometries, displaying highly similar differences in hemodynamics (Supplementary Fig. S8) with reduced computational costs for the CFD simulation.

To create the volume mesh for the fluid domain, the lumen surface mesh was triangulated, capped and exported to Ansys Fluent (Ansys Inc., Canonsburg, PA, USA, 2021R2). In Fluent, the surface mesh was further refined to 0.4 mm. For the boundary layer, 4 layers of prism elements with an aspect ratio of 1 were employed with an increase of 20% in element size for each layer. The element size equaled 0.4 mm for the first layer and 0.7 mm for the last layer. Tetrahedral elements were used to mesh the interior. An increase in element size of 20% was prescribed with a maximum element size of 1.4 mm. A generic, time-varying flow profile (Supplementary Fig. S2) was prescribed at the inlet of the fluid domain. The maximum, mean and minimum flow equaled 4.9, 0.95 and − 0.4 L/min, respectively. A Poiseuille profile was assumed to describe the velocity over the radius of the vessel.^1^ The Carreau model was used to model the shear-thinning behavior of blood and a no-slip condition was assigned to the lumen wall.^25^ A 3-element Windkessel model, consisting of a characteristic impedance (Z), peripheral resistance (R) and arterial compliance (C), was used to prescribe the pressure at the outlets of the fluid domain, using the outlet flow. The Windkessel parameters were determined using the mean pressure and flow, and the geometrical and mechanical properties of the outlet, and were optimized by iteratively adjusting the peripheral resistance and compliance to match the patient-specific blood pressure (BP). For each patient, the BP as measured at the clinical visit closest to the date of the CT scan was used in the simulations, since no BP measurement was performed at the date of the CT scan. The brachial BP was converted into the abdominal aortic BP, as explained in Fonken *et al*.^13^ The inlet flow was assumed to be divided over the iliac arteries according to $\overline{{q_{{\text{i}}} }} = \overline{{q_{{{\text{in}}}} }} \frac{{a_{{\text{i}}}^{3} }}{{a{\text{L}}^{3} + a_{{\text{R}}}^{3} }}$^19^ with subscript *i* indicating the left (*L*) or right (*R*) iliac artery. The mean pressure was assumed to be equal in both iliac arteries. A heart rate of 75 beats per minute was used and three cardiac cycles were simulated. A more detailed description of the CFD model is found in Fonken *et al*.^13^

### Result Analyses

To investigate the spread in parametric inlet and bifurcation characteristics, the obtained parametric geometries were compared to the patient-specific geometries by calculating the percentual differences in inlet and bifurcation parameters.

For each patient, three CFD simulations were performed: one with the original, patient-specific geometry, one where the inlet was replaced with a parametric geometry and one where the bifurcation was replaced with a parametric geometry. The flow properties in the aneurysm region resulting from the simulations with parametric inlet and bifurcation meshes were compared to the flow properties in the aneurysm region of the patient-specific geometry. To this end, the systolic Wall Shear Stress ($\hbox {WSS}_{\text{sys}}$), time-averaged Wall Shear Stress (TAWSS), and Oscillatory Shear Index (OSI) of the last cardiac cycle were evaluated.^13^ For each quantity ($\phi$), the values resulting from the patient-specific (*PS*) simulation and the parametric (inlet or bifurcation) simulation were interpolated to a similar grid in the aneurysm region and the point-wise difference ($\delta _{\phi }$) and percentual point-wise difference ($\delta p_{\phi }$) were calculated according to:

4 $$\begin{aligned} \begin{aligned}&\delta _{\phi } = \phi _{\text{parametric}} - \phi _{\text{PS}}\\&\delta p_{\phi } = \frac{\delta _{\phi }}{\bar{\phi }_{\text{PS}}} \cdot 100\% \end{aligned} \end{aligned}$$

With $\bar{\phi }_{\text{PS}}$ the average quantity value for the patient-specific (PS) geometry.

## Results

**TABLE 1:** Inlet and bifurcation parameter values calculated from the patient-specific dataset (median and IQR) and the resulting parameters used to generate parametric inlet and bifurcation geometries.

	Dataset (Median ± IQR)	Parametric	Description
$L_{{{\text{in}}}}$ (cm)	5.4 ± 4.7	5.4	Inlet length
$\Delta$e $({\text {m}}^{-1})$	23.6 ± 17.9	23.6	Increase rate in eccentricity towards bifurcation curve
$\Delta r_{{\text {L}}}$ $({\text {m}}^{-1})$	0.29 ± 0.27	0.29	Increase rate in long radius towards bifurcation curve
$\Delta r_{{\text {S}}}$ $({\text {m}}^{-1})$	0.48 ± 0.44	0.48	Decrease rate in short radius towards bifurcation curve
$e_{{\text {bif}}}$ (–)	0.98 ± 0.02	0.98	Eccentricity of the bifurcation curve
$R_{{\text {R,L}}}$ (–)	1.09 ± 0.37	1.00	Ratio between left and right iliac radius
$R_{{\text {1,end}}}$ (–)	1.09 ± 0.19	1.00	Ratio between first and last iliac radius
$R_{{\text {r,L,bif}}},r_{{\text {I}}}$ (–)	0.41 ± 0.09	0.41	Ratio between long radius of the bifurcation curve and iliac radius
$L_{{\text {I}}}$ (cm)	2.75 ± 0.08	2.75	Length of iliac arteries
$\alpha _{{\text {R,L}}}$ ($^{\circ }$)	47.5 ± 16.5	50.0	Bifurcation angle

### Parametric Inlet and Bifurcation Geometries

**FIGURE 3:**
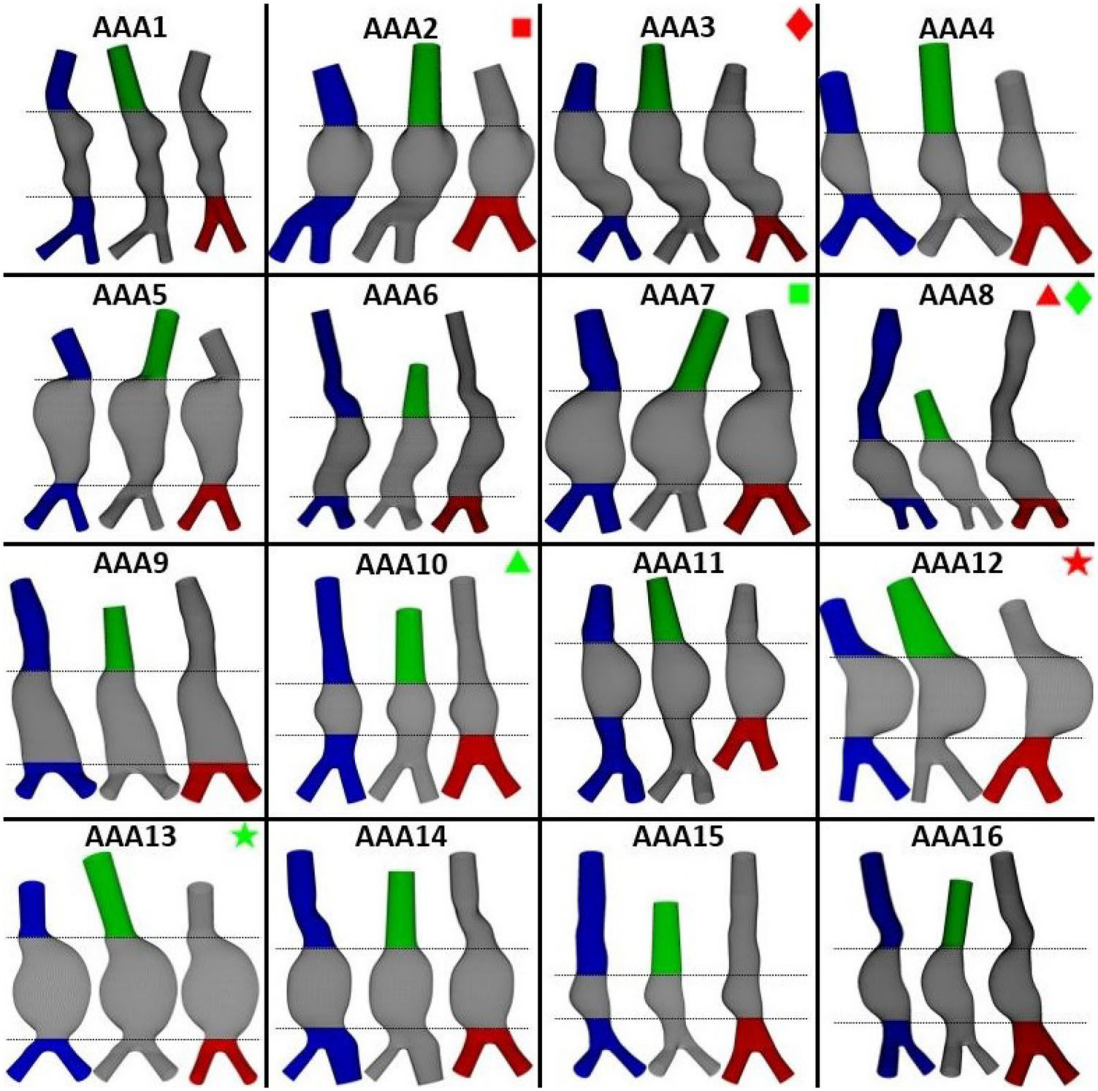
Overview of the original, patient-specific geometries (blue), geometries with parametric inlet (green) and geometries with parametric bifurcation (red) for all patients. The aneurysm region is identical for all meshes.The colored symbols indicate which geometries will be highlighted in the other figures.

**FIGURE 4:**
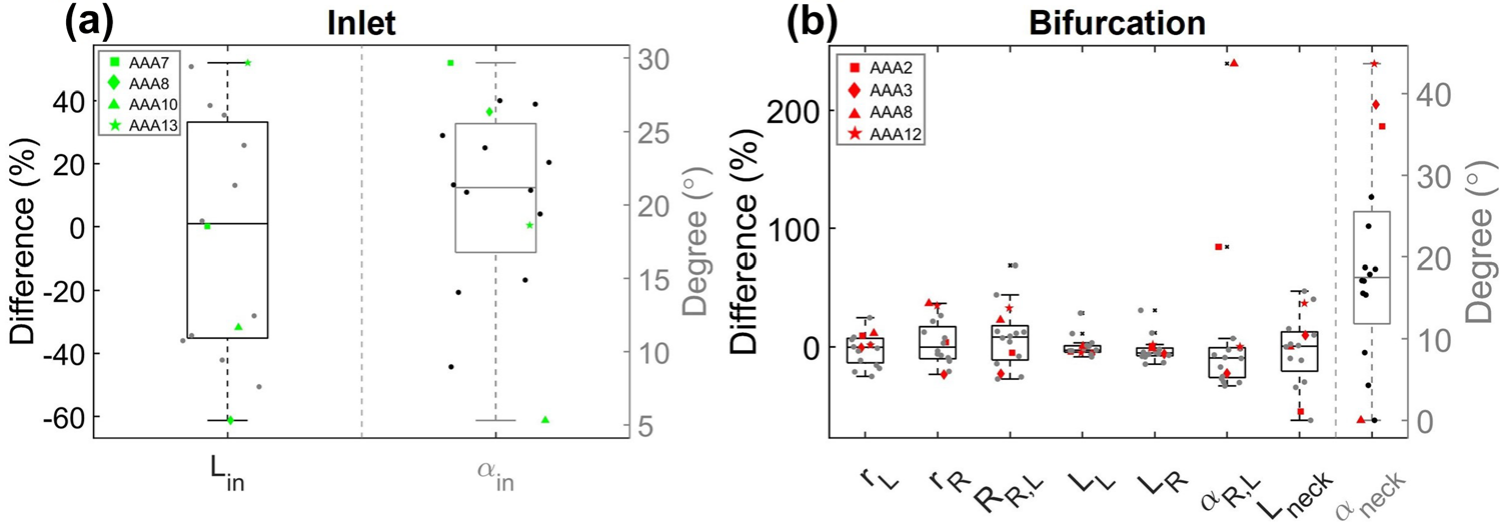
(a) visualization of the percentual difference in inlet length $(\hbox {L}_{\text{in}})$ and the angle ($\alpha _{\text{in}}$) between the patient-specific and parametric inlet. (b) Visualization of the percentual difference in left iliac radius and length $(\hbox {r}_{\text{L}}$ and $\hbox {L}_{\text{L}})$, right iliac radius and length $(\hbox {r}_{\text{R}}$ and $\hbox {L}_{\text{R}})$, ratio between right and left iliac radii $(\hbox {R}_{\text{R,L}})$, bifurcation angle ($\alpha _{\text{R,L}}$), distal neck length $(\hbox {L}_{\text{neck}})$ and distal neck angle ($\alpha _{\text{neck}}$) between the patient-specific and parametric geometry. In each plot, the values for 4 patients are highlighted (see legend). The values for all other patients are plotted as dots.

The median and IQR values of the inlet and bifurcation parameters for all 16 patient-specific geometries are listed in Table 1. For simplicity, the ratio between the left and right iliac radius and the ratio between the first and last iliac radius were set to 1 to create the parametric bifurcation, since the median values for both ratios was close to 1. The bifurcation angle was chosen to be 50$^{\circ }$, closely resembling the median value of the dataset and in agreement with literature.^9,26^ For all other parameters, the median values as calculated from the dataset were used.

The resulting parametric inlet and bifurcation geometries for all patients are shown in Fig. 3, alongside the patient-specific geometries. As summarized in Fig. 4a, significant differences in inlet length and direction between the patient-specific and parametric inlet geometries were detected. The difference in inlet direction was quantified by calculating the angle between the patient-specific and parametric inlet centerline ($\alpha _{\text{in}}$). The median difference in inlet length was approximately zero (1.0%) and a large spread in differences was observed (IQR 68.4%). When looking at the absolute difference, a median deviation of 34.9% (2.1 cm) was found. These results display that parametric geometries with shorter and longer inlet lengths were generated. The angle between the patient-specific and parametric inlet ranged from 5.3$^{\circ }$ to 29.7$^{\circ }$ (IQR 8.8$^{\circ }$), with a median of 21.2$^{\circ }$, indicating that parametric inlet geometries with varying orientations were obtained.

For the parametric bifurcation, considerable differences in distal neck length, bifurcation angle, iliac radii, and the ratio between iliac radii were observed between the patient-specific and parametric bifurcation geometries, as summarized in Fig. 4b. Furthermore, the distal neck direction differed noticeably, which was quantified by calculating the angle between the patient-specific and parametric distal neck centerline ($\alpha _{\text{neck}}$). For two patients (AAA8 and AAA9), no distal neck was added, since the last AAA curve was located directly proximal to the iliac arteries, resulting in no differences in distal neck length and angle. The median differences in left and right iliac radius and distal neck length were approximately zero. However, the median values of the absolute differences were 8.8%, 11.5% and 13.4%, respectively. Furthermore, a large spread in values can be observed (IQR 15.0%, 15.8% and 32.6%, respectively). For the ratio between the left and right iliac radius, a median difference of 8.3% was found, indicating that, most often, the ratio for the parametric bifurcation was higher compared to the ratio for the patient-specific bifurcations. However, the boxplot shows a large spread (IQR of 29.0%), indicating that parametric geometries with smaller and larger ratios, compared to the patient-specific geometry, were obtained. The left and right iliac length have slightly decreased in the parametric bifurcation (median of − 2.9 and − 5.2%, respectively). The spread in iliac length was small (IQR of 5.5 and 6.5% for left and right, respectively), as also observed in Table 1. In most cases, the parametric geometry had a decreased bifurcation angle, with a median of − 9.4%. A large spread (IQR 25.3%) and one extreme outlier (AAA8) can be observed in Fig. 4b. The boxplot indicates that parametric geometries with increased and decreased bifurcation angles were obtained. Lastly, the distal neck angle had a median value of 17.5$^{\circ }$ with a large spread (IQR 13.7$^{\circ }$), indicating that varying distal neck orientations were obtained in the parametric bifurcation.

In both subfigures of Fig. 4, the values of four patients are highlighted. The results for these patients will be discussed in more detail in Sects. 4.2 and 4.3. These patients were selected to reflect the large variety in (differences between) patient-specific and parametric inlet and bifurcation geometries.

### Influence of Inlet Geometry

**FIGURE 5:**
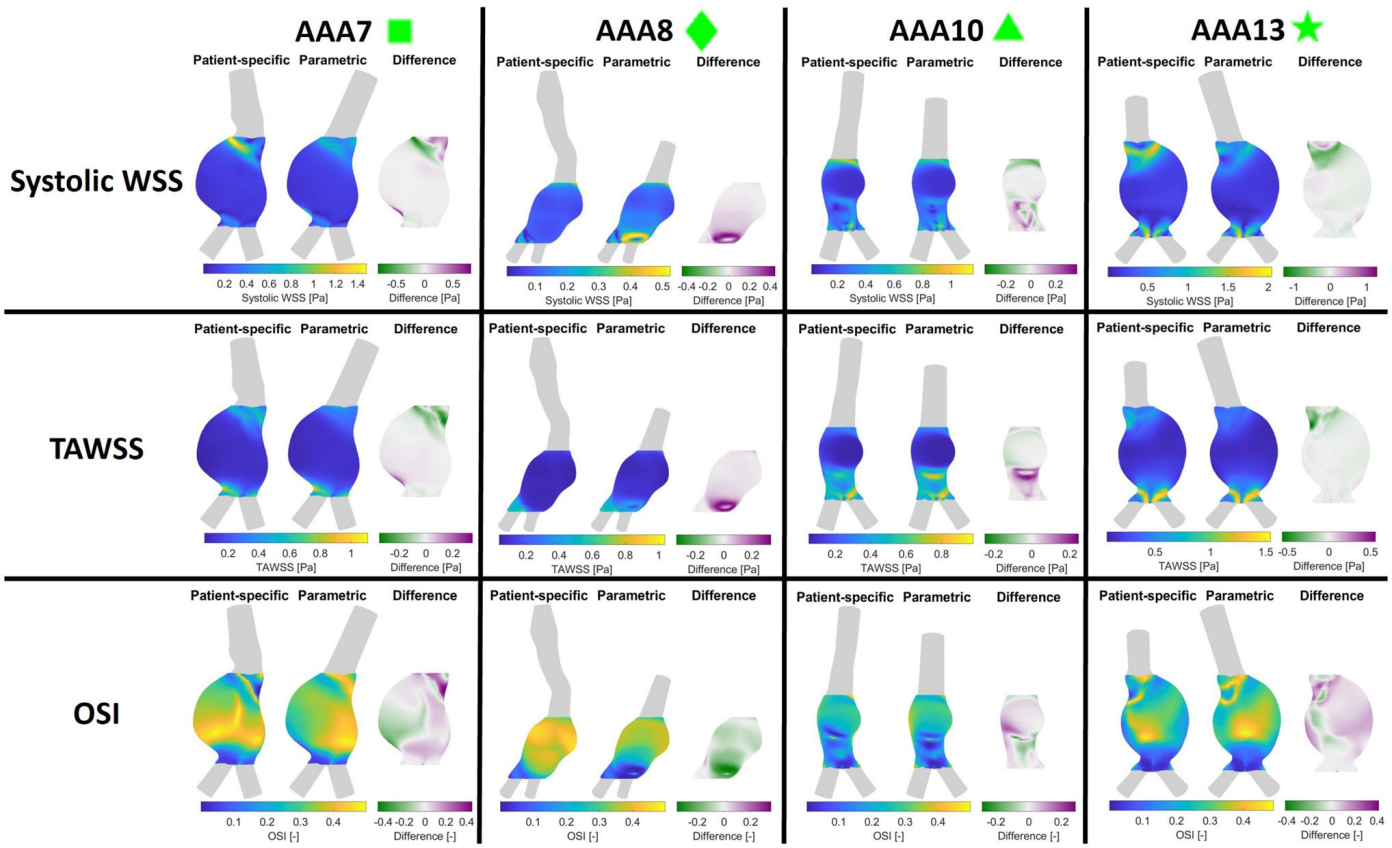
Visualization of the $\hbox {WSS}_{\text{sys}}$, TAWSS, and OSI resulting from CFD simulations for the patient-specific and parametric inlet geometries of patients AAA7, AAA8, AAA10 and AAA13.

The CFD simulations for both the patient-specific and parametric inlet geometries were executed successfully for all 16 patients. Supplementary Fig. S3 visualizes the $\hbox {WSS}_{\text{sys}}$, TAWSS and OSI values in the aneurysm region and their point-wise differences for all patients. The results for patients AAA7, AAA8, AAA10 and AAA13 are shown in Fig. 5. As explained in Sect. 4.1, these patients were selected based on their varying inlet geometries and are therefore representative for the complete dataset. Considerable differences in hemodynamics in the complete AAA region were observed for all patients (Fig. 5). For the $\hbox {WSS}_{\text{sys}}$ and TAWSS, the major differences were observed in the proximal region when the length of the parametric inlet was similar or increased w.r.t. the length of the patient-specific inlet (AAA7 and AAA13). When the parametric inlet length was decreased (AAA8 and AAA10), the largest differences occur distally in the AAA region. The OSI pattern in the aneurysm has changed noticeably between the patient-specific and parametric geometries, although this difference was relatively small for the patient with the smallest angle between the patient-specific and parametric inlet (AAA10).

**FIGURE 6:**
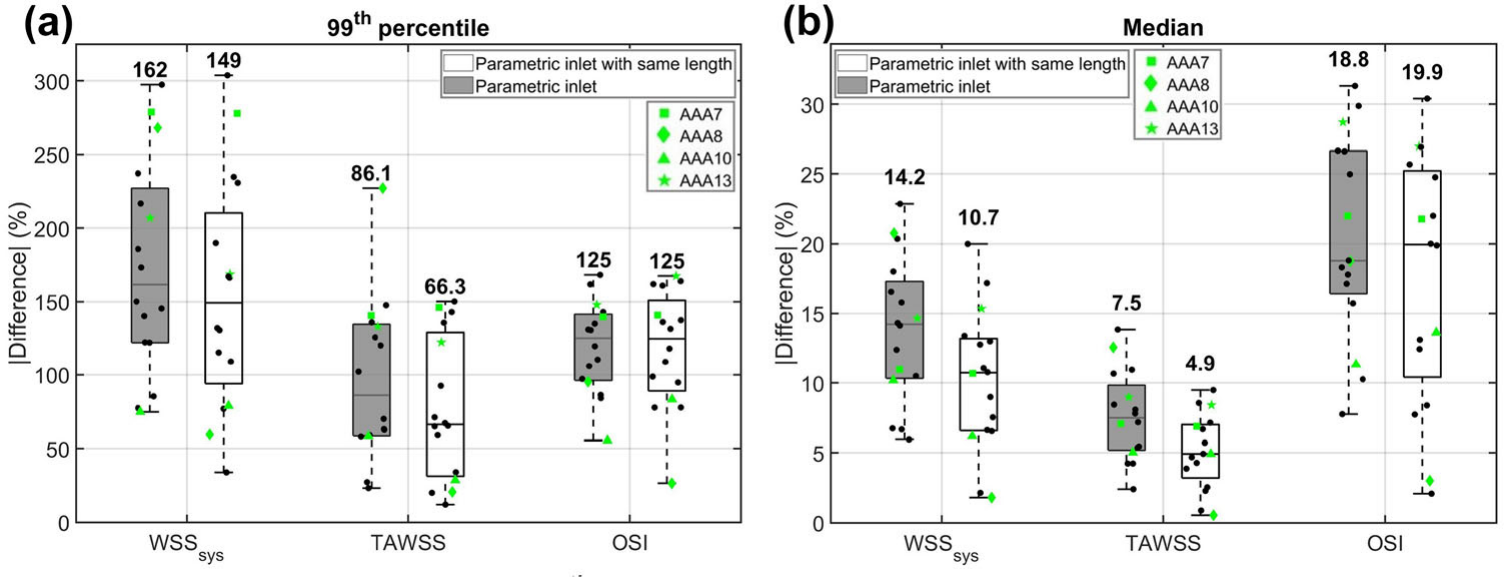
Visualization of the $99{\text{th}}$ percentile (a) and median (b) absolute percentual point-wise difference in $\hbox {WSS}_{\text{sys}}$, TAWSS and OSI for the parametric inlet geometries (dark) and parametric inlet geometries with the same length as the patient-specific geometry (light). The bold number on top of each boxplot indicates the median. The values for the selected patients are highlighted (see legend) and the values for the other patients are plotted as black dots.

An additional analysis showed no significant correlations $(\hbox {R}^{2}$ $\le$ 0.37) between the inlet angle ($\alpha _{\text{in}}$) or difference in inlet length $(\hbox {L}_{\text{in}})$ and the median differences in hemodynamic quantities (Fig. S4). This could be explained by the fact that both parameters were altered simultaneously. To further investigate the importance of both parameters separately, another set of simulations was executed with parametric inlet geometries with the same inlet length as found in the patient-specific geometry (Fig. S5). Figure 6 summarizes the $99{\text{th}}$ percentile and median absolute percentual point-wise difference in $\hbox {WSS}_{\text{sys}}$, TAWSS and OSI for the parametric inlet geometries and parametric inlet geometries with the same length. These boxplots indicate that large percentual differences, up to 300%, were observed when the patient-specific inlet was replaced by a parametric one. When only the inlet angle was taken into account (inlet with the same length), the median value of the $99{\text{th}}$ percentile difference in $\hbox {WSS}_{\text{sys}}$ and TAWSS decreased from 162 to 149% and from 86.1 to 66.3%, respectively. The median values of the absolute median difference decreased from 14.2 to 10.7% $(\hbox {WSS}_{\text{sys}})$ and from 7.5 to 4.9% (TAWSS). For the OSI, the median of the $99{\text{th}}$ percentile differences was not affected when only the inlet angle was taken into account. Furthermore, the median value of the median absolute difference was slightly increased (from 18.8 to 19.9%), although the spread was larger.

### Influence of Bifurcation Geometry

**FIGURE 7:**
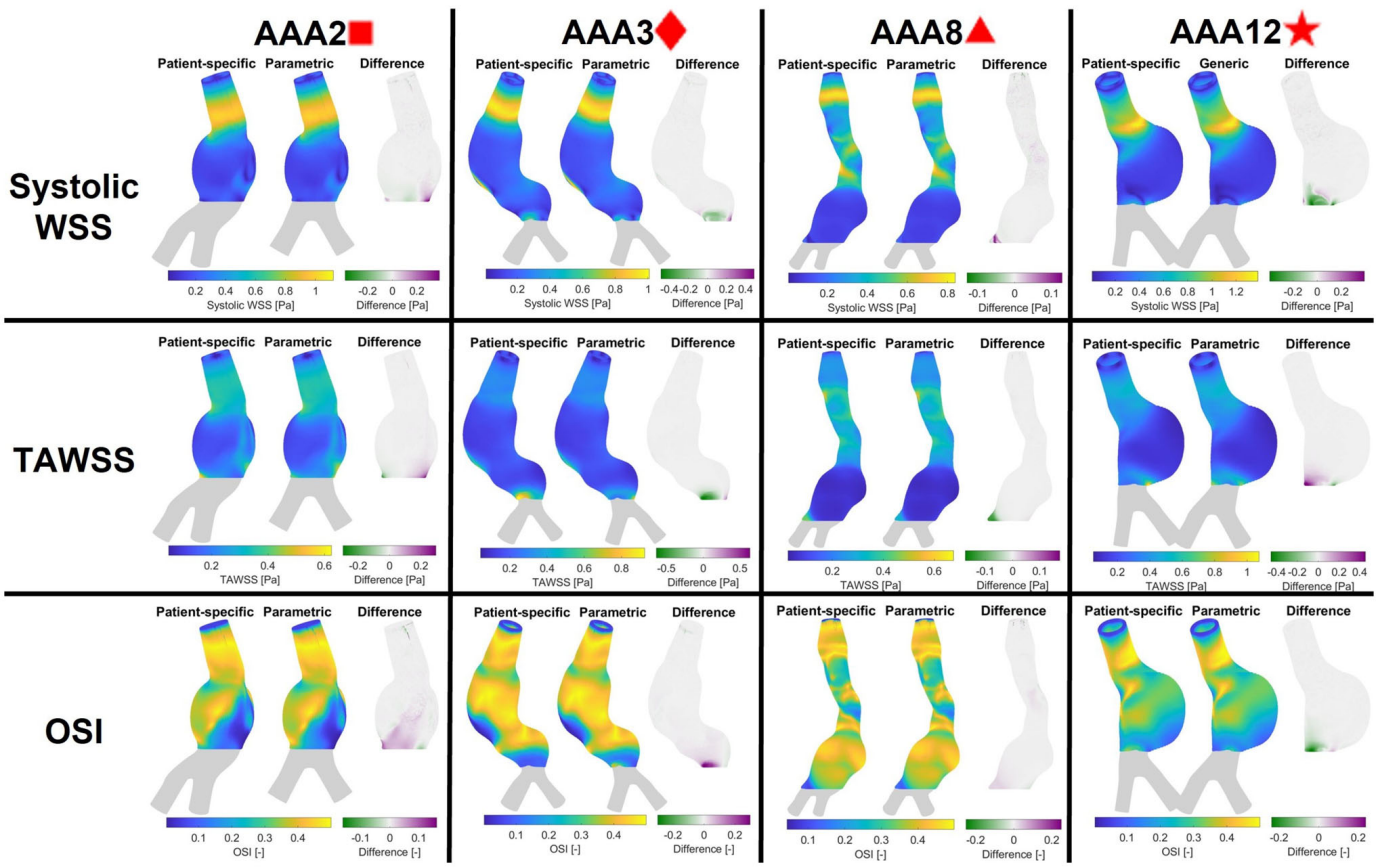
Visualization of the $\hbox {WSS}_{\text{sys}}$, TAWSS, and OSI resulting from CFD simulations for the patient-specific and parametric bifurcation geometries of patients AAA2, AAA3, AAA8 and AAA12.

Again, for all 16 patients included in this study, the CFD simulations with the parametric bifurcation geometry were executed successfully. For all patients, the $\hbox {WSS}_{\text{sys}}$, TAWSS, and OSI values in the aneurysm region for both geometries are visualized, together with their point-wise differences, in Supplementary Fig. S6. In Fig. 7, the results for patients AAA2, AAA3, AAA8 and AAA12 are displayed. These patients were selected based on the large differences between their patient-specific and parametric bifurcation geometry (Sect. 4.1). In all cases, the largest differences were observed in the distal part of the aneurysm region. In the remainder of the AAA region, the point-wise differences were small.

**FIGURE 8:**
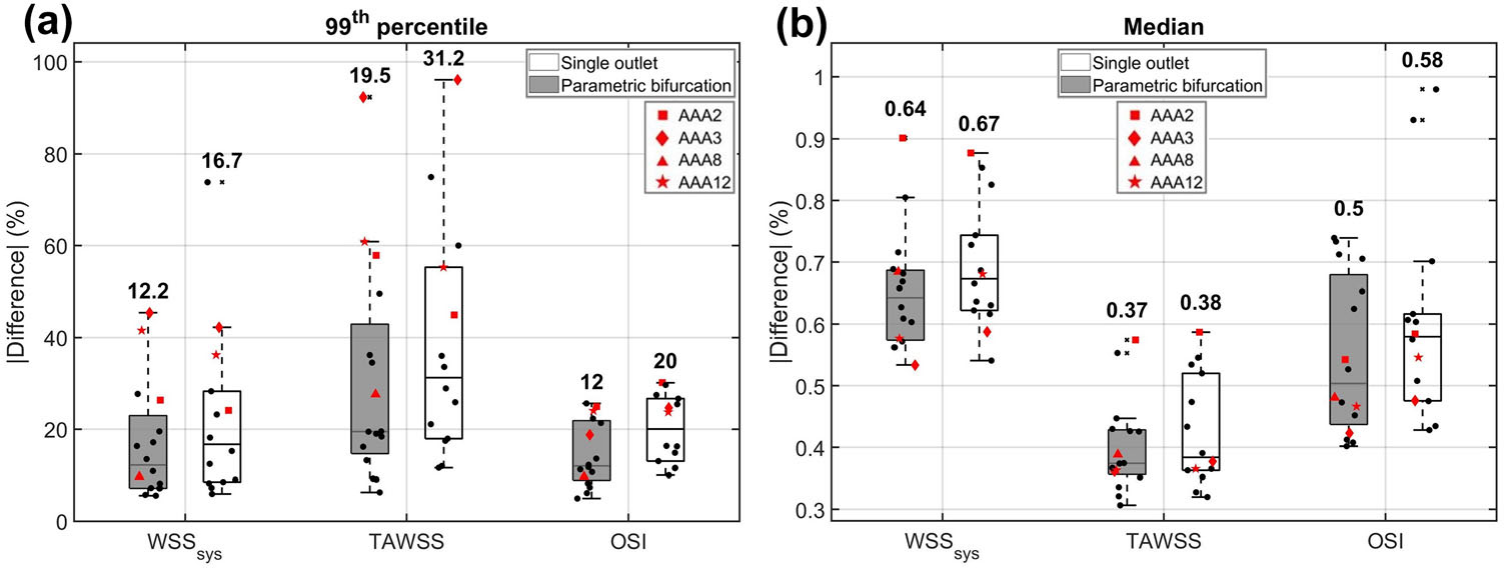
Visualization of the $99{\text{th}}$ percentile (a) and median (b) absolute percentual point-wise difference in $\hbox {WSS}_{\text{sys}}$, TAWSS and OSI for the parametric bifurcation geometries (dark) and single outlet geometries (light). The bold number on top of each boxplot indicates the median. The values for the selected patients are highlighted (see legend) and the values for the other patients are plotted as black dots.

This observation can be confirmed with Fig. 8, which summarizes the $99{\text{th}}$ percentile and median absolute percentual point-wise difference in $\hbox {WSS}_{\text{sys}}$, TAWSS and OSI for the parametric bifurcation geometry. All medians of the $99{\text{th}}$ percentile difference values were below 20% (12.2%, 19.5% and 12% for the $\hbox {WSS}_{\text{sys}}$, TAWSS and OSI, respectively). For all hemodynamic quantities and all patients, the median absolute difference was below 1%, with median values of 0.64%, 0.37% and 0.5% for the $\hbox {WSS}_{\text{sys}}$, TAWSS and OSI, respectively.

To investigate the feasibility of further simplifying the outlet geometry, an additional analysis was performed, in which CFD simulations were executed with geometries with a single outlet instead of a bifurcation (Fig. S7). For two patients (AAA8 and AAA9), the shape of the last AAA contour closely resembled the lemniscate shape of the bifurcation contour, making it impossible to generate a geometry with a single outlet without altering the AAA geometry. Therefore, these patients were excluded from the comparison between the parametric bifurcation and the single outlet. As shown in Fig. 8, the median value of the $99{\text{th}}$ percentile increases from 12.2 to 16.7% ($\hbox {WSS}_{\text{sys}}$), 19.5 to 31.2% (TAWSS) and 12 to 20% (OSI) when a single outlet was used instead of a parametric bifurcation. Furthermore, slight increases in median values were observed (0.64 to 0.67% for $\hbox {WSS}_{\text{sys}}$, 0.37 to 0.38% for TAWSS and 0.5 to 0.58% for OSI). These results indicate that replacing the patient-specific bifurcation by a single outlet results in larger percentual point-wise differences compared to replacing it by a parametric bifurcation.

## Discussion

In this study, a framework was developed to add parametric inlet and bifurcation geometries to the abdominal aortic aneurysm geometry, employing parameters of the AAA geometry and dataset statistics. The impact of replacing the patient-specific inlet and bifurcation geometries, acquired using CT scans, by parametric geometries was evaluated by examining the differences in hemodynamics in the aneurysm region.

As shown in Sect. 4.1, parametric inlet and bifurcation geometries for all patients were created successfully using the AAA geometry and dataset statistics. Figures 3 and 4 illustrate the feasibility of adding a parametric inlet and bifurcation to an AAA segmentation and reveal a large variety in differences in parameters between the patient-specific and parametric geometries, except for the length of the iliac arteries. This small range in iliac length was also observed in the dataset statistics (Table 1) and can be explained by the fact that the part of the iliac arteries that moves out of plane was removed from the Hemodyn segmentation (Sect. 3.1). This is a disadvantage of the current framework, in which contours on specified *Z*-coordinates were defined, and could be resolved by creating geometries based on contours perpendicular to the centerline. However, this would require significant changes to the modeling framework, which would only be necessary for CT data, since the iliac arteries are usually not visible on 3D+t US data. Since the framework is envisioned to be applied to 3D+t US data and the iliac arteries can be elongated to mitigate the effect of differences in iliac length (Fig. S1), the framework was not adapted for this study.

As observed in Figs. 5 and 6, considerable differences in the complete aneurysm region were observed when the patient-specific inlet geometry was replaced by a parametric one. The median values for the absolute median difference in $\hbox {WSS}_{\text{sys}}$, TAWSS and OSI equalled 14.2% ,7.5% and 18.8%, respectively. When only the inlet angle was taken into account, the median values of the $99{\text{th}}$ percentile and median difference for the $\hbox {WSS}_{\text{sys}}$ and TAWSS decreased by 8 to 35%. However, the effect on the differences in OSI were neglectable. These results indicate that it is not feasible to replace the patient-specific inlet geometry by a parametric one, since this causes significant differences in hemodynamic quantities in the AAA region. Furthermore, it can be concluded that the inlet angle is the most important inlet parameter, since the inlet length has a relatively small effect on the differences in hemodynamic values, especially for the OSI.

In a single 3D+t ultrasound acquisition, the inlet geometry often cannot be captured. Multiperspective ultrasound could resolve this limitation. With this technique, proximal and distal 3D+t US acquisitions could be obtained and fused to obtain ultrasound data with an extended FOV, including the inlet geometry.^31^ However, the fusing of multiple 3D+t US acquisitions is not part of the current workflow and is not trivial, since both spatial and temporal registration need to be performed. Alternatively, a longitudinal 2D ultrasound scan proximal to the aneurysm could be acquired and registered to the (single) 3D+t ultrasound acquisition. This method would yield the inlet geometry in a single plane, which should be extended to 3D. A disadvantage of this approach is the fact that assumptions about the inlet geometry are necessary to obtain the 3D geometry, which might introduce inaccuracies. An advantage of this approach is that the 2D ultrasound scan proximal to the aneurysm region could simultaneously be used to extract the patient-specific velocity profile using ultrasound Doppler, which can be used to further personalize the envisioned FSI modeling framework as described in Fonken *et al*.^13^

Apart from some outliers, the obtained differences in hemodynamics between the patient-specific bifurcation geometry and the parametric bifurcation were small, with median differences below 1%, as discussed in Sect. 4.3. As observed in Figs. 7 and S6, the major differences in hemodynamics were observed in the distal part of the aneurysm region, which was expected, since this part of the AAA geometry is closest to the bifurcation geometry. Studies of Darling *et al*.^7^ and Boyd *et al*.^3^ have shown that aneurysm rupture generally occurs in the proximal or middle section of the aneurysm. Therefore, the hemodynamics in the distal part of the aneurysm are believed to be less relevant to aneurysm development and rupture. Hence, the observed differences in the distal part of the AAA are deemed acceptable for this application.

When the outlet geometry was further simplified by replacing the parametric bifurcation with a single outlet, the percentual point-wise differences increased. Although the obtained differences in hemodynamics for the single outlet are still small, these results indicate that the parametric bifurcation is preferred to limit the differences. Furthermore, no single outlet geometry could be obtained for two patients, in which the aneurysm region extended until the bifurcation, whereas a parametric bifurcation could be obtained for all patients. Adding a bifurcation instead of a single outlet to an aneurysm geometry induces a bit more complexity in the meshing framework and results in a slightly larger mesh and longer simulation time. However, the decrease in differences in hemodynamics for the parametric bifurcation outweighs these disadvantages. Furthermore, for patients with high echogenicity, in which certain bifurcation parameters, such as distance to the bifurcation and the iliac radii, can be measured, the bifurcation geometry could be further personalized using the proposed framework, by simply altering the parameters.

No correlations were found between differences in bifurcation parameters and differences in hemodynamics, indicating that no dominant bifurcation parameter was found. This could be explained by the fact that all bifurcation parameters were altered simultaneously. To investigate the influence of a specific bifurcation parameter on the hemodynamics, this parameter should be altered while keeping all other parameters constant. In this study, such a parametric study was not deemed necessary, since the differences between the patient-specific and parametric bifurcation geometries were generally very small.

CFD simulations were employed to obtain the hemodynamics in the AAA region. As discussed in the introduction (Sect. 2), the rigid wall assumption in CFD simulations yields an overestimation of WSS compared to FSI simulations.^18^ However, only the differences in hemodynamics were examined in this study, not the absolute values. For a single patient, CFD and FSI simulations were performed for the patient-specific and parametric bifurcation geometries. The obtained differences in AAA hemodynamics were highly similar for the CFD and FSI simulations (Supplementary Fig. S8). Since the computational costs of FSI simulations were increased by a factor 23 compared to CFD simulations and only minor discrepancies in differences in hemodynamics were found, the use of CFD simulations in this study can be justified.

The Womersley number is approximately 12 in our simulations, so the flow is formally inertia dominated. Therefore, a Womersley flow profile might be a better assumption than the Poiseuille profile. However, since the Womersley profile might slightly differ for different inlet geometries, whereas the Poiseuille profile is identical in all cases, the Poiseuille profile was used in this study. Furthermore, since this study investigated the influence of differences in geometry, the employed velocity profile is of lesser interest and its influence on the differences in hemodynamics between the geometries is expected to be less important. Finally, only the higher harmonics of the inlet flow profile cause the Womersley profile to deviate from the Poiseuille profile. Since the fundamental frequency is significantly large with respect to the higher harmonics, only moderate deviations of the Poiseuille profile are expected. In a future study, the impact of the velocity profile on the AAA hemodynamics could be investigated.

Although the obtained framework is envisioned to be applied on 3D+t US data, CT data was used to evaluate the influence of the limited FOV, since the patient-specific inlet and bifurcation geometries cannot be extracted with a (single) 3D+t US acquisition.^28^ The use of 3D+t US will induce uncertainty in the segmentation, due to the lower contrast compared to CT. However, in previous research, 3D+t US-based segmentations were compared to CT-based segmentations and showed good correspondence. Furthermore, 3D+t US-based CSS simulations resulted in comparable stresses as CT-based CSS simulations.^29^ In future research, the framework first needs to be extended to accurately extract the inlet geometry, for example with the use of an additional 2D US acquisition proximal to the aneurysm. Subsequently, 3D+t US-based FSI simulations should be compared to CT-based FSI simulations to quantify the uncertainty of using 3D+t US on full FSI outcome.

To conclude, this study showed that it is not feasible to add a parametric inlet geometry to an AAA geometry, since replacing the patient-specific inlet geometry by a parametric one caused significant differences in the hemodynamics in the aneurysm region. Future research should investigate the possibilities of extending the proximal field-of-view of 3D+t US, using multiperspective 3D+t US imaging or an additional 2D (Doppler) US acquisition. However, this study did demonstrate the feasibility of adding a parametric bifurcation geometry to an aneurysm geometry, using dataset statistics and parameters of the AAA geometry. Replacing the patient-specific bifurcation by a parametric version resulted in only small differences in systolic WSS, TAWSS and OSI values, with the largest differences observed in the distal part of the AAA, where rupture generally does not occur. These results clearly show that the inlet geometry has a larger effect on AAA hemodynamics than the bifurcation geometry. After extending the proximal FOV, the obtained framework can be used to obtain AAA geometries from 3D+t US data for FSI simulations, despite the absence of the aorto-iliac bifurcation. Furthermore, the bifurcation geometry can easily be personalized, if patient-specific bifurcation parameters are available.

## Supplementary Information

Below is the link to the electronic supplementary material.

Supplementary file1 (PDF 9455 kb)
