# Supplementary material for: The Impact of a Limited Field-of-View on Computed Hemodynamics in Abdominal Aortic Aneurysms: Evaluating the Feasibility of Completing Ultrasound Segmentations with Parametric Geometries
Source: Ann Biomed Eng. 2023 Jan 28;51(6):1296–309. doi: 10.1007/s10439-022-03133-6 (PMC10172266; doi:10.1007/s10439-022-03133-6)
Supplement: Supplementary file 1 — Supplementary file1 (PDF 9455 kb) [file 10439_2022_3133_MOESM1_ESM.pdf]

Supplementary material

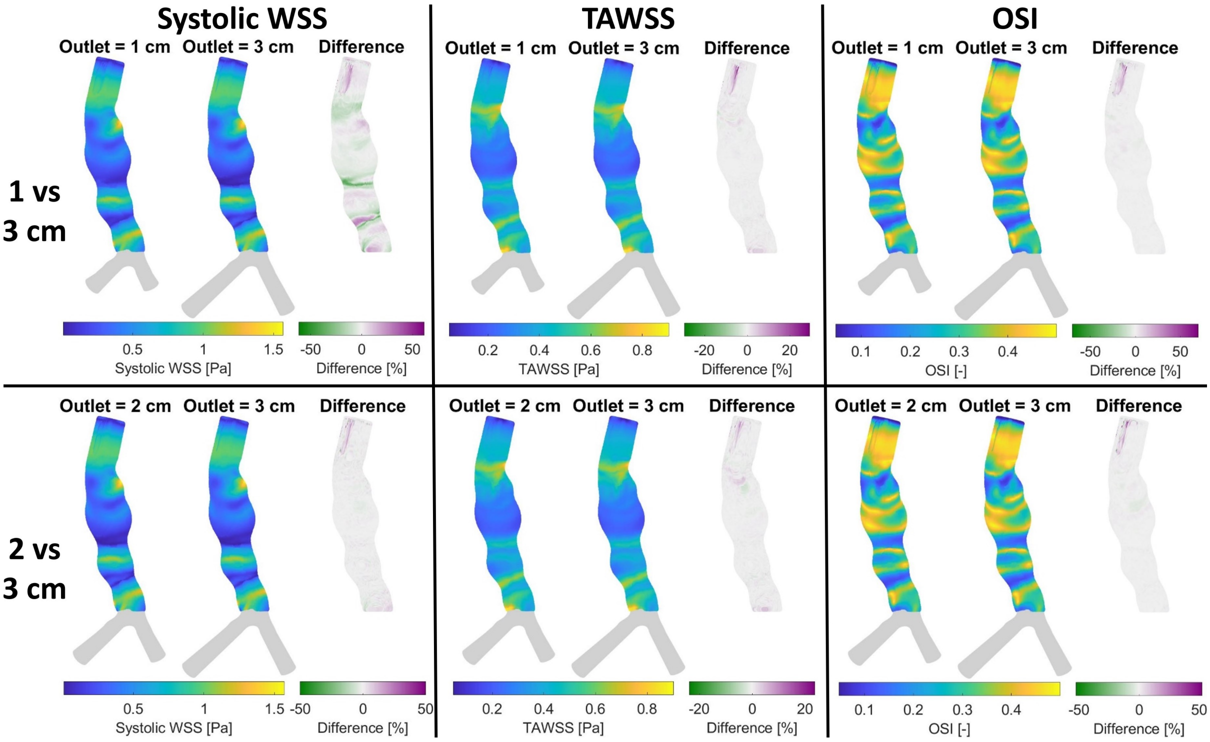

Figure S1: Visualization of the systolic WSS, TAWSS and OSI resulting from CFD simulations for the patient-specific geometry of patient AAA1 with iliac elongations of 1, 2 or 3 cm. Based on these results, an iliac elongation of 2 cm was added in the patient-specific framework in Sec. 3.1.)

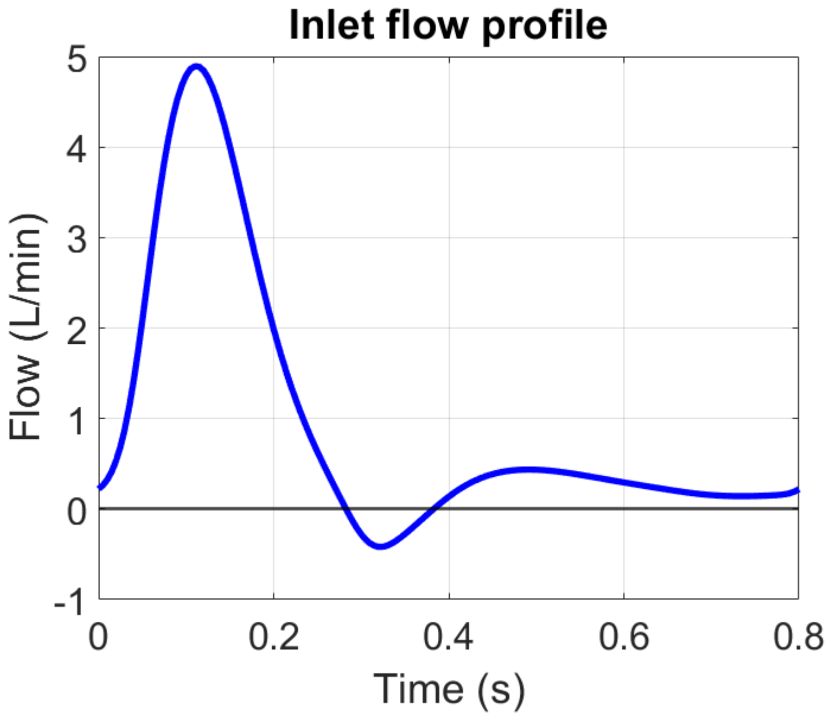

Figure S2: The inlet flow waveform as prescribed at the inlet.

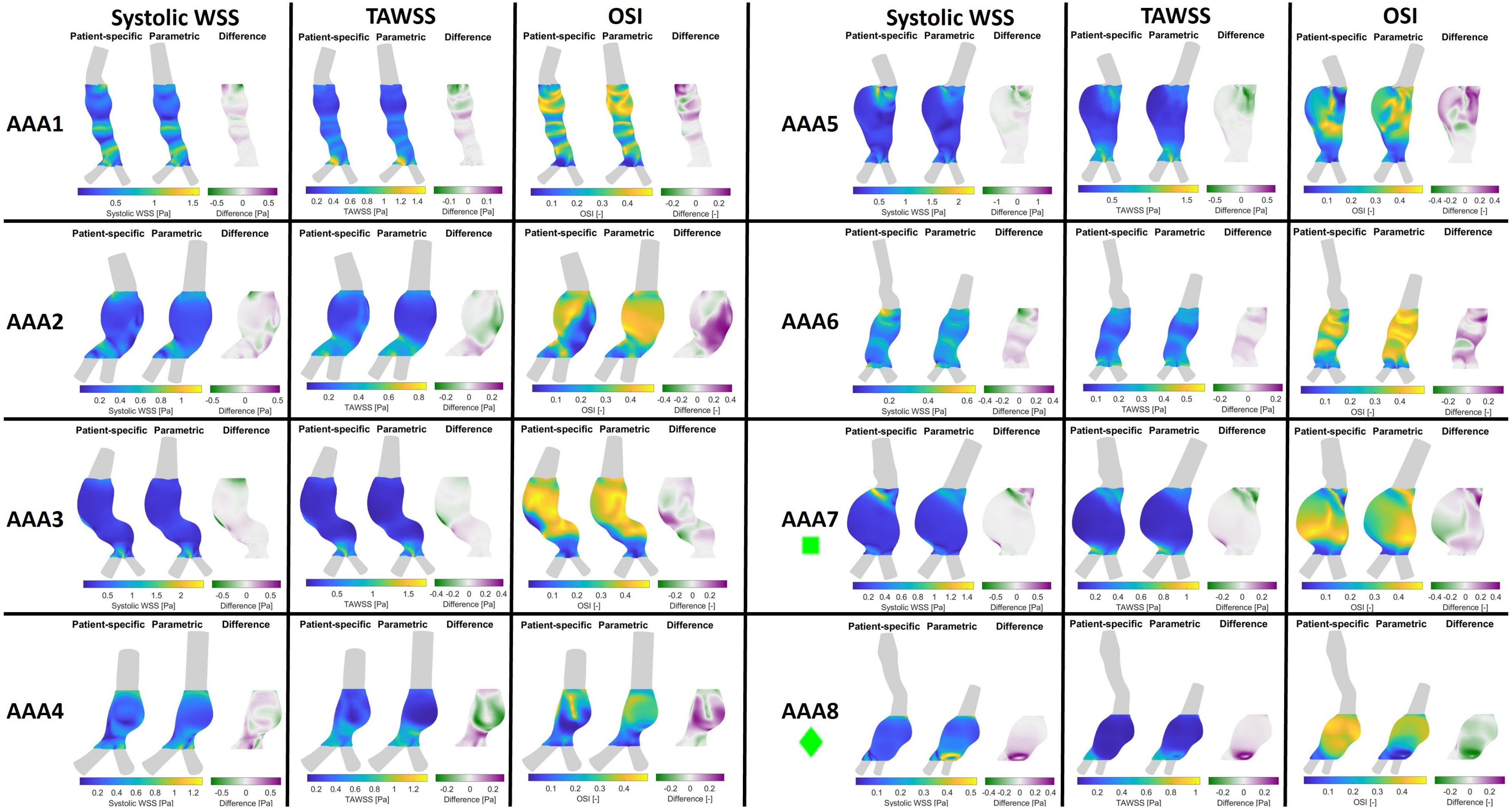

Figure S3: Systolic WSS, TAWSS and OSI resulting from CFD simulations for the patient-specific and parametric inlet geometries for all patients. Figure continues on next page.

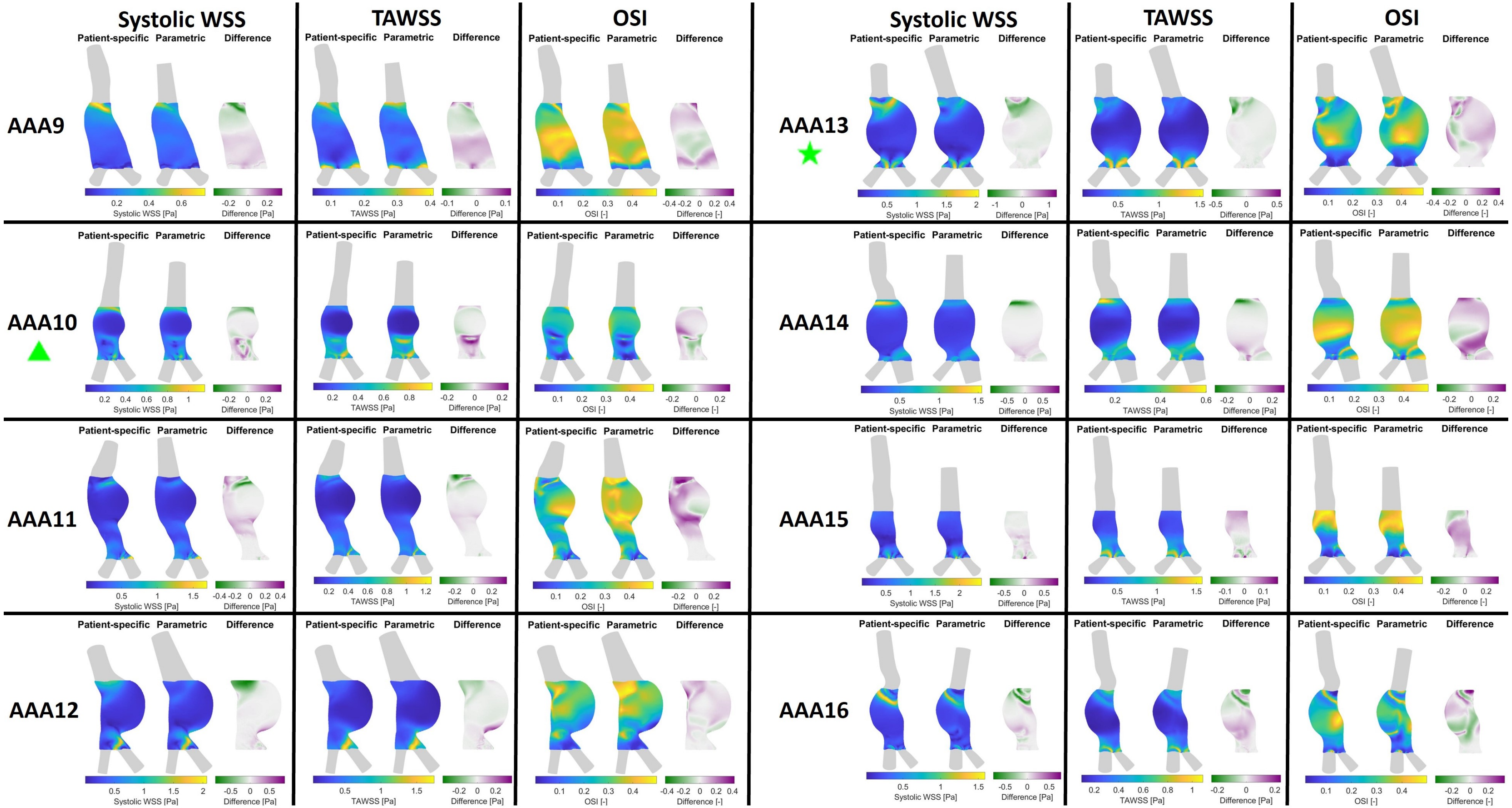
Systolic WSS
TAWSS
OSI
Patient-specific
Parametric
Difference
AAA9
AAA10
AAA11
AAA12
AAA13
AAA14
AAA15
AAA16
Systolic WSS [Pa]
TAWSS [Pa]
OSI [-]
Difference [Pa]
Difference [-]

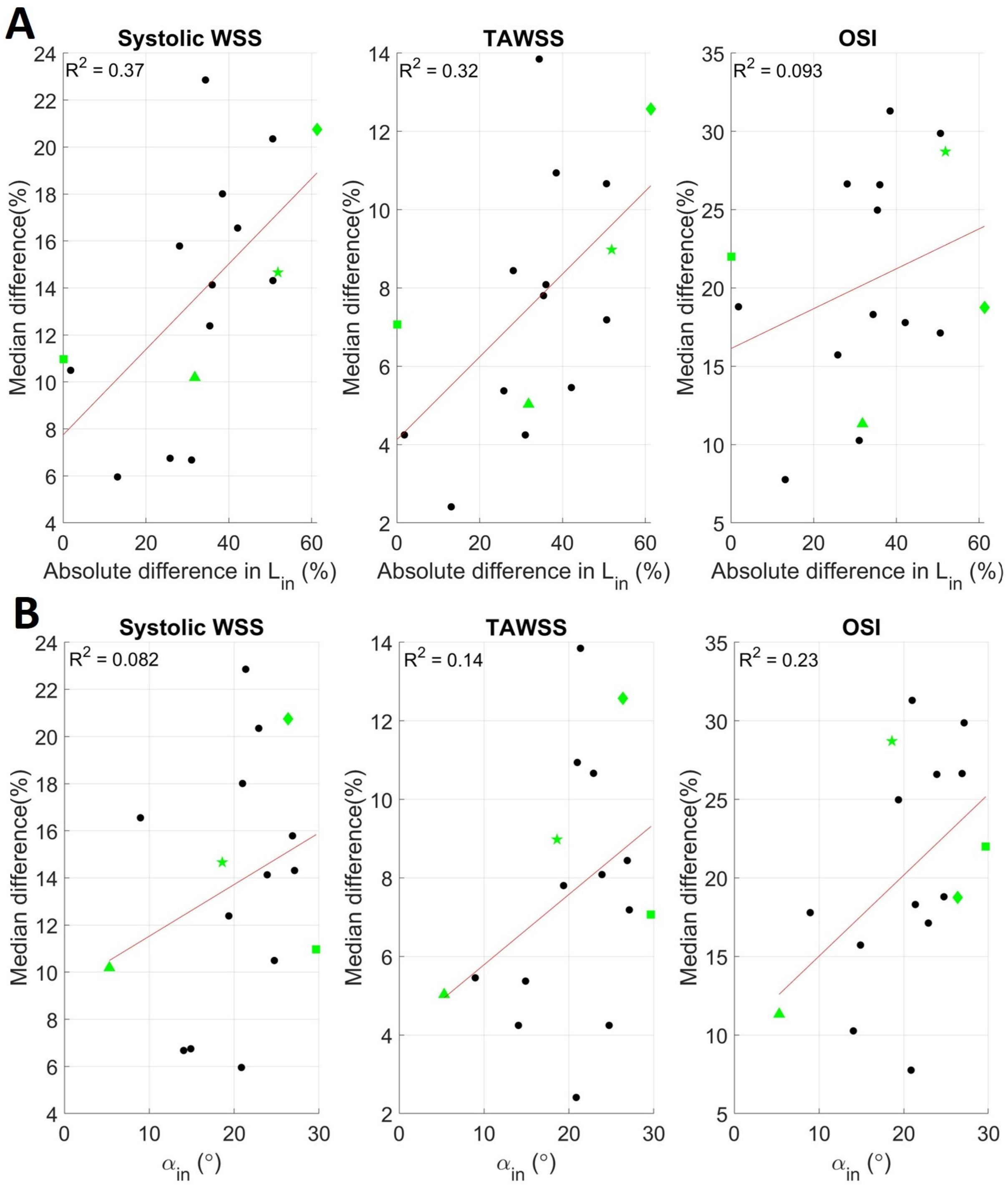

Figure S4: Scatter-plots of the median differences in WSS_{sys}, TAWSS and OSI as function of (A) the absolute difference in inlet length or (B) the angle between patient-specific and parametric inlet. The red solid line indicates the linear fit and the R^2 is indicated in the left corner of each plot.

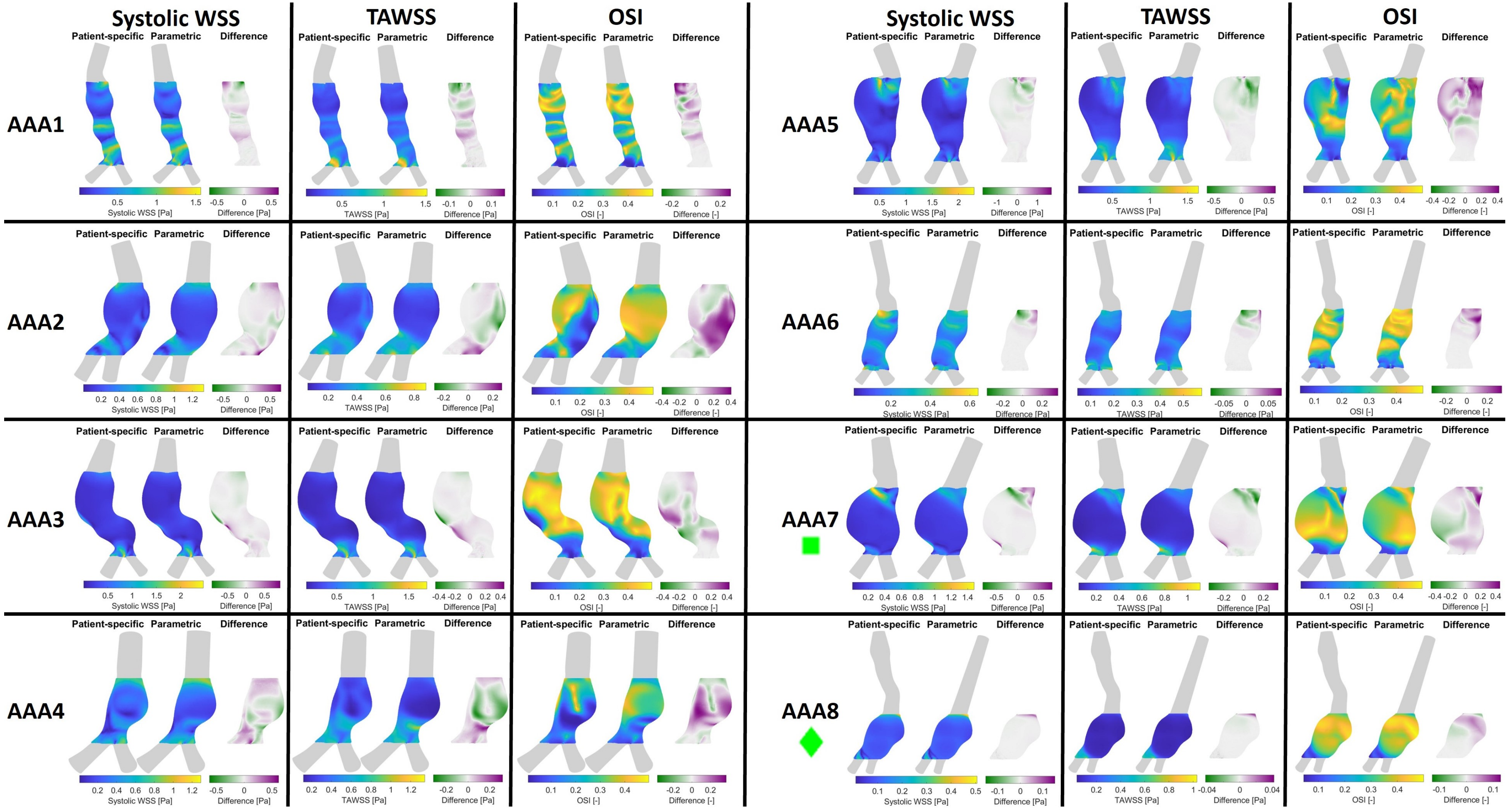

Figure S5: Systolic WSS, TAWSS and OSI resulting from CFD simulations for the patient-specific and parametric inlet geometries with the same inlet length for all patients. Figure continues on next page.

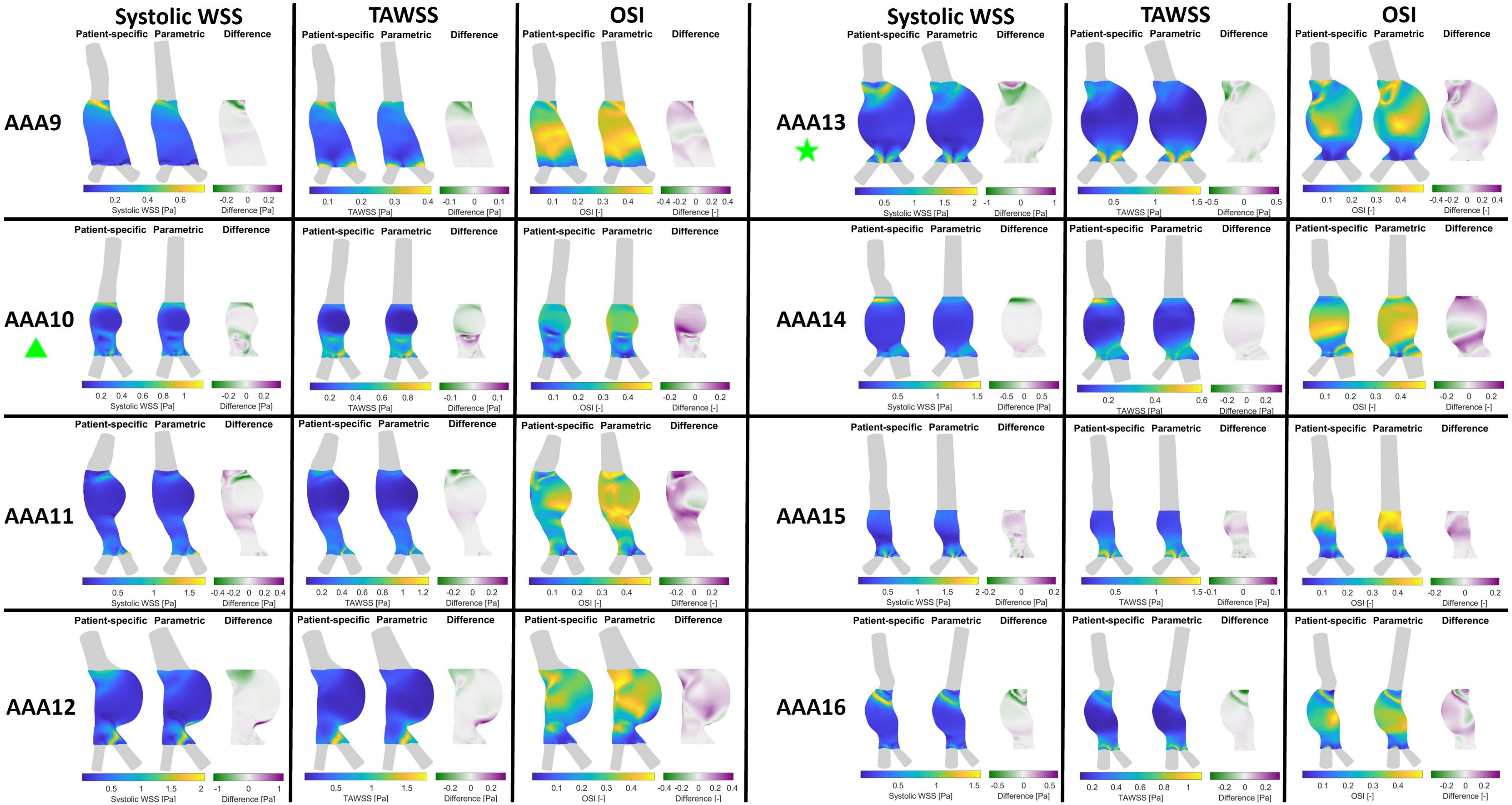
Systolic WSS
TAWSS
OSI
Systolic WSS
TAWSS
OSI
Patient-specific
Parametric
Difference
AAA9
AAA10
AAA11
AAA12
AAA13
AAA14
AAA15
AAA16
Systolic WSS [Pa]
TAWSS [Pa]
OSI [-]
Difference [Pa]
Difference [-]

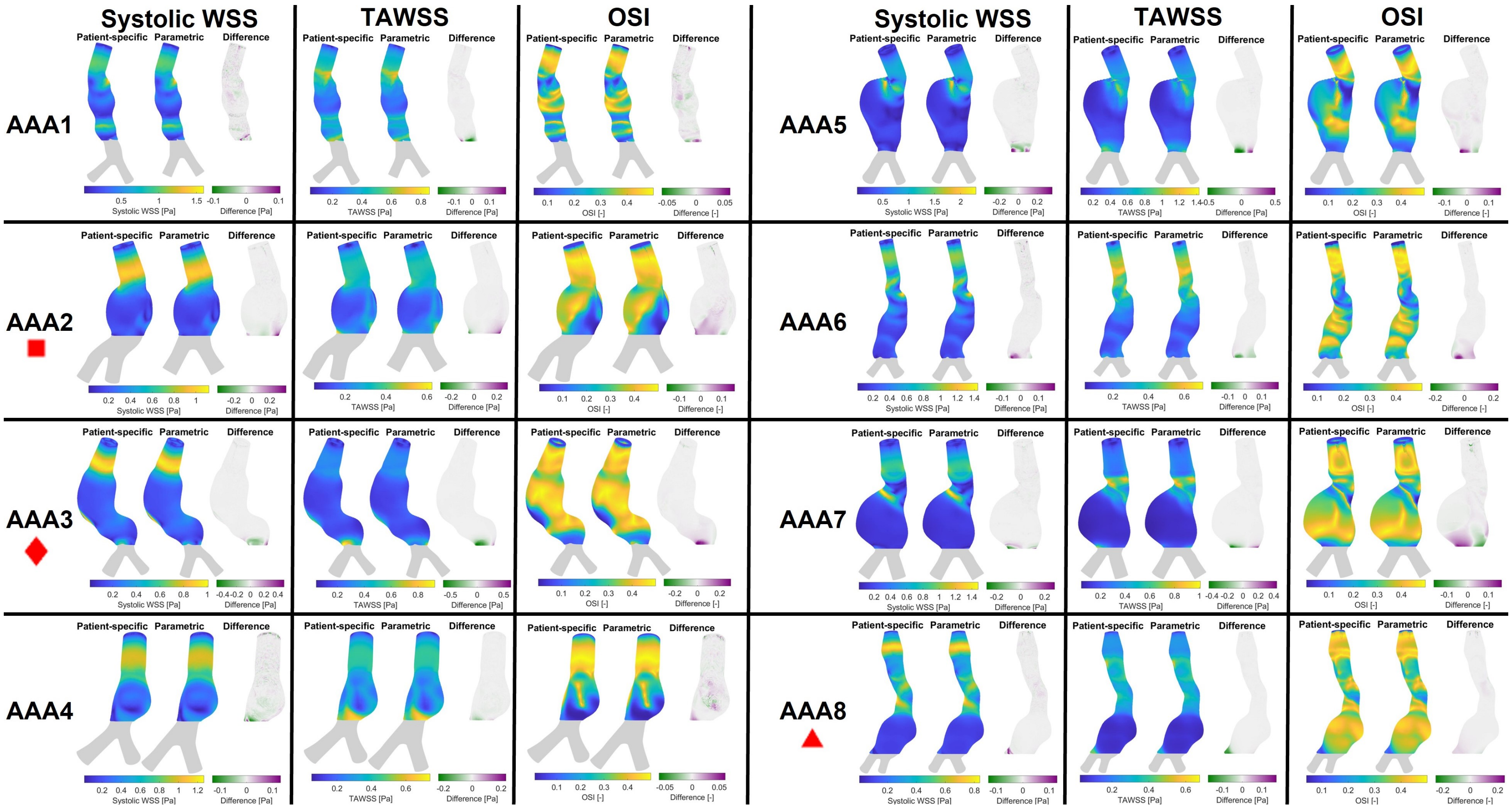

Figure S6: Systolic WSS, TAWSS and OSI resulting from CFD simulations for the patient-specific and parametric bifurcation geometries for all patients. Figure continues on next page.

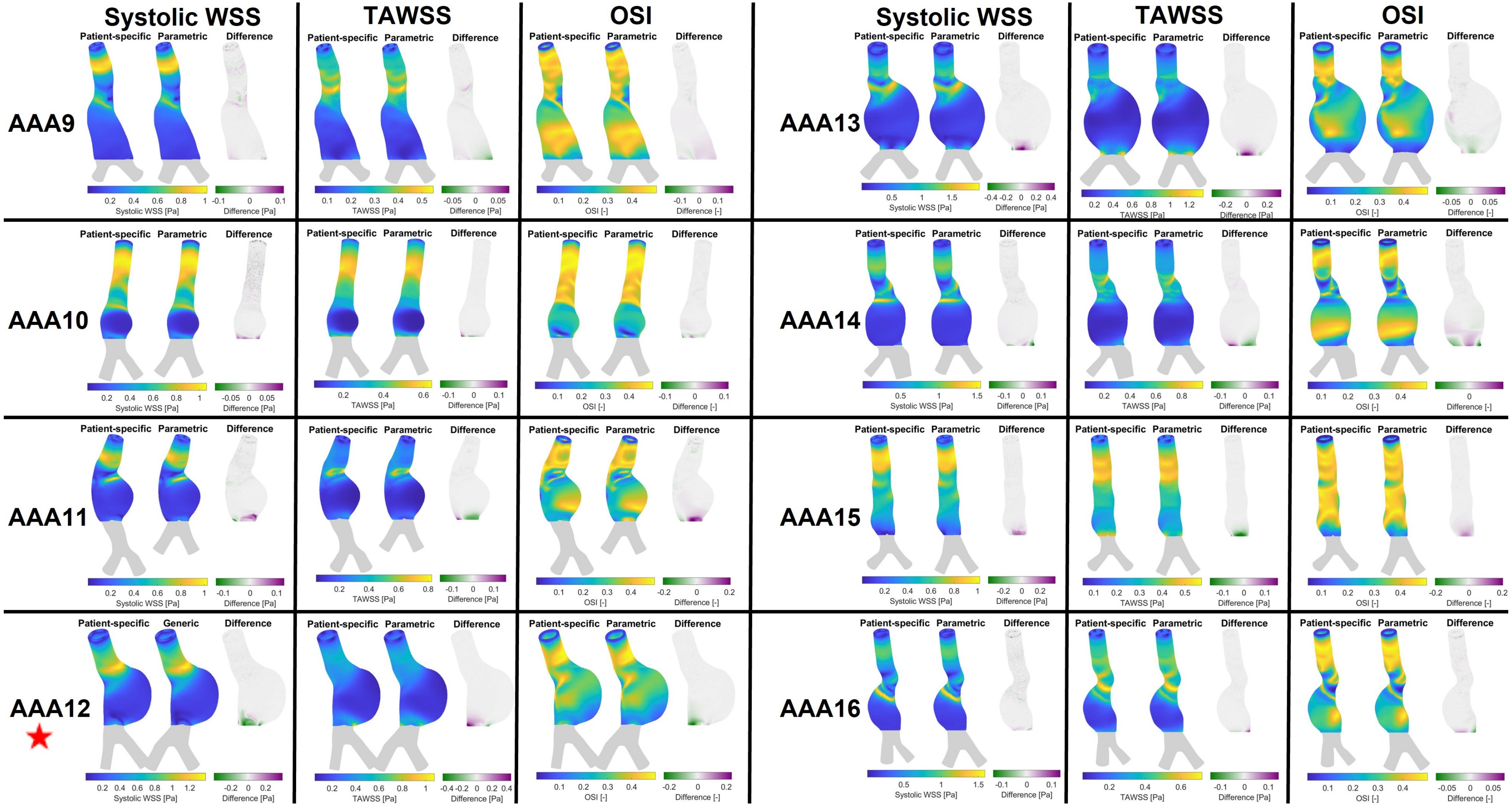
Systolic WSS
TAWSS
OSI
Patient-specific
Parametric
Generic
Difference
AAA9
AAA10
AAA11
AAA12
AAA13
AAA14
AAA15
AAA16
Systolic WSS [Pa]
TAWSS [Pa]
OSI [-]
Difference [Pa]
Difference [-]

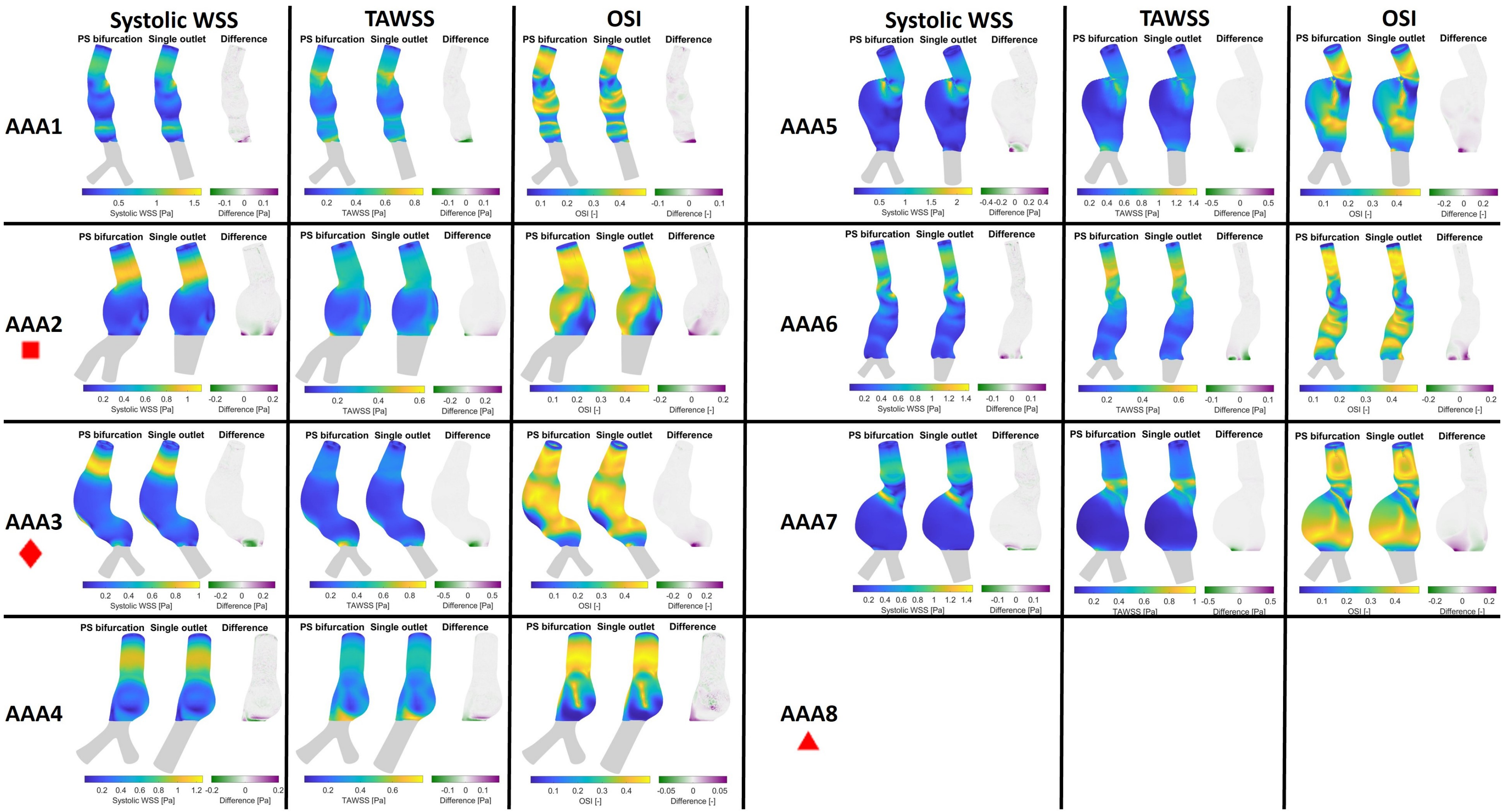

Figure S7: Systolic WSS, TAWSS and OSI resulting from CFD simulations for the patient-specific bifurcation and single outlet geometries all patients, except AAA8 and AAA9. Figure continues on next page.

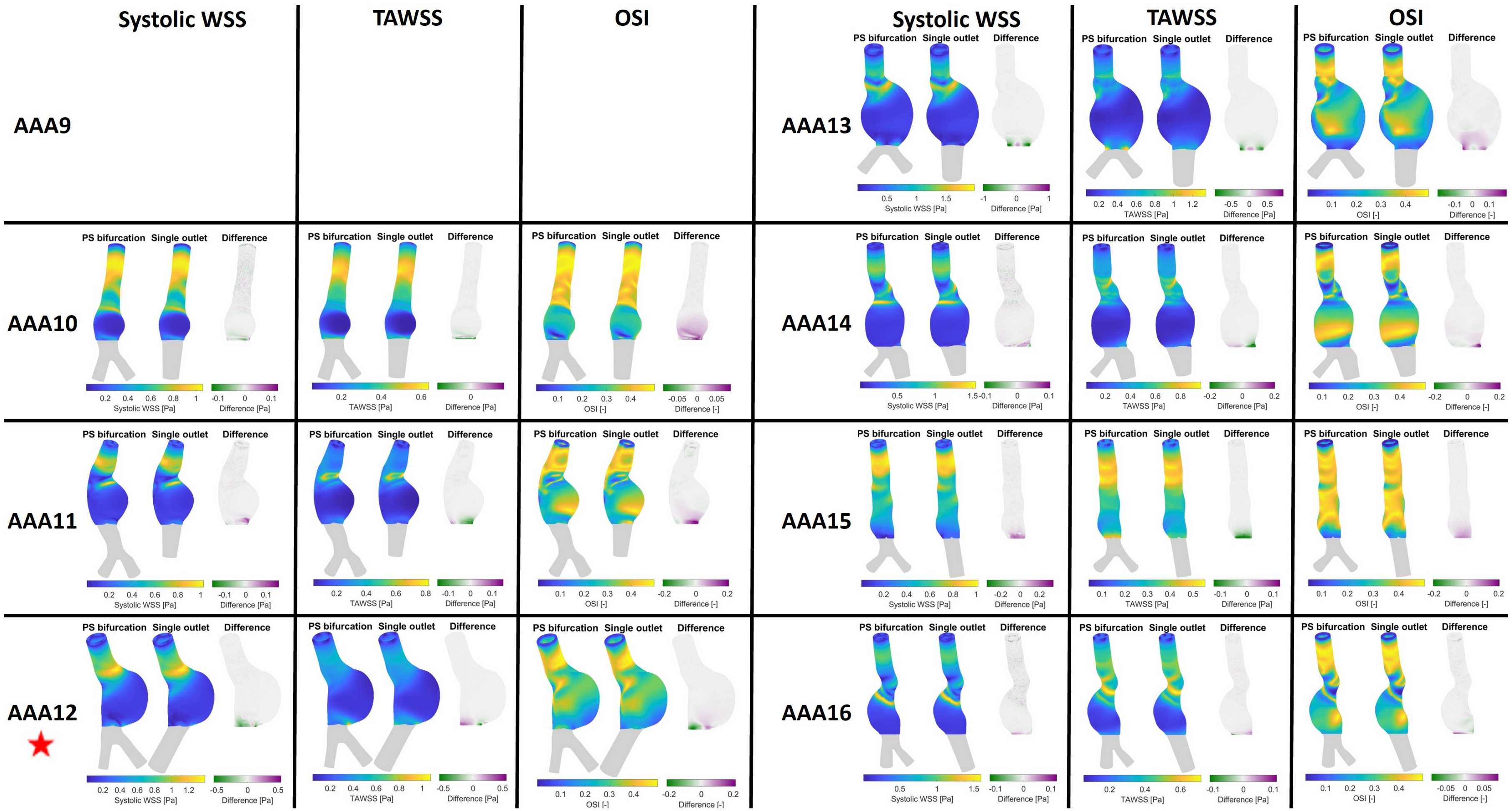
Systolic WSS
TAWSS
OSI
PS bifurcation
Single outlet
Difference
AAA9
AAA10
AAA11
AAA12
AAA13
AAA14
AAA15
AAA16
Systolic WSS [Pa]
TAWSS [Pa]
OSI [-]
Difference [Pa]
Difference [-]

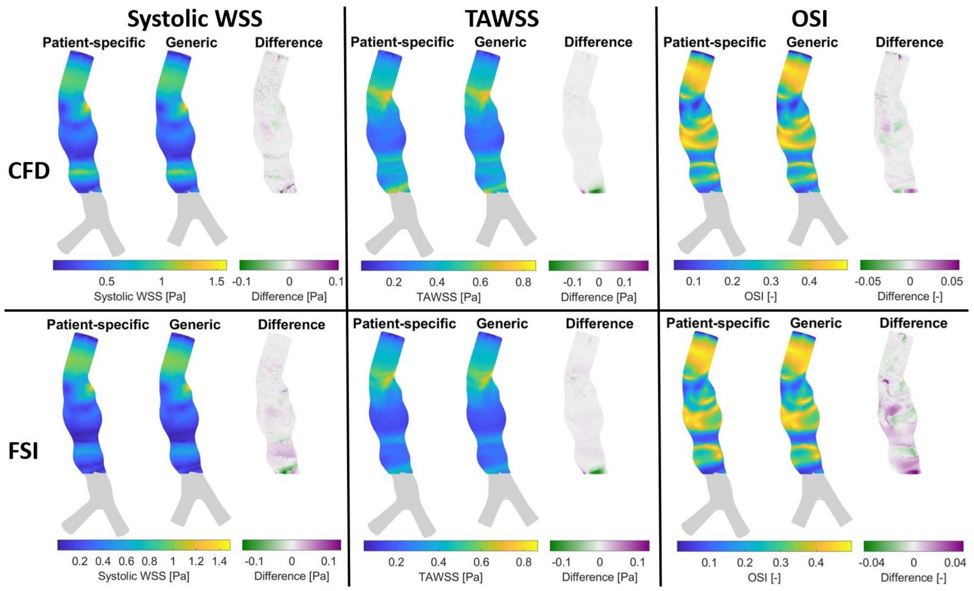

Figure S8: Comparison between FSI and CFD models to quantify the differences in AAA hemodynamics between the patient-specific and parametric bifurcation geometries for patient 1. The WSS_{sys}, TAWSS and OSI values in the AAA region and their differences are visualized for CFD (top) and FSI (bottom) simulations.
